# Targeting HIF-1**α** abrogates PD-L1–mediated immune evasion in tumor microenvironment but promotes tolerance in normal tissues

**DOI:** 10.1172/JCI150846

**Published:** 2022-05-02

**Authors:** Christopher M. Bailey, Yan Liu, Mingyue Liu, Xuexiang Du, Martin Devenport, Pan Zheng, Yang Liu, Yin Wang

**Affiliations:** 1 Division of Immunotherapy, Institute of Human Virology, Department of Surgery and Comprehensive Cancer Center, University of Maryland School of Medicine, Baltimore, Maryland, USA.; 2 Institute of Biomedical Sciences, The George Washington University School of Medicine & Health Sciences, Washington DC, USA.; 3 Key Laboratory of Infection and Immunity of Shandong Province & Department of Immunology, School of Basic Medical Sciences, Shandong University, Jinan, China.; 4 OncoC4, Inc, Rockville, Maryland, USA.

**Keywords:** Therapeutics, Cancer immunotherapy

## Abstract

A combination of anti–CTLA-4 plus anti–PD-1/PD-L1 is the most effective cancer immunotherapy but causes high incidence of immune-related adverse events (irAEs). Here we report that targeting of HIF-1**α** suppressed PD-L1 expression on tumor cells and tumor-infiltrating myeloid cells, but unexpectedly induced PD-L1 in normal tissues by an IFN-**γ**–dependent mechanism. Targeting the HIF-1**α**/PD-L1 axis in tumor cells reactivated tumor-infiltrating lymphocytes and caused tumor rejection. The HIF-1**α** inhibitor echinomycin potentiated the cancer immunotherapeutic effects of anti–CTLA-4 therapy, with efficacy comparable to that of anti–CTLA-4 plus anti–PD-1 antibodies. However, while anti–PD-1 exacerbated irAEs triggered by ipilimumab, echinomycin protected mice against irAEs by increasing PD-L1 levels in normal tissues. Our data suggest that targeting HIF-1**α** fortifies the immune tolerance function of the PD-1/PD-L1 checkpoint in normal tissues but abrogates its immune evasion function in the tumor microenvironment to achieve safer and more effective immunotherapy.

## Graphical abstract

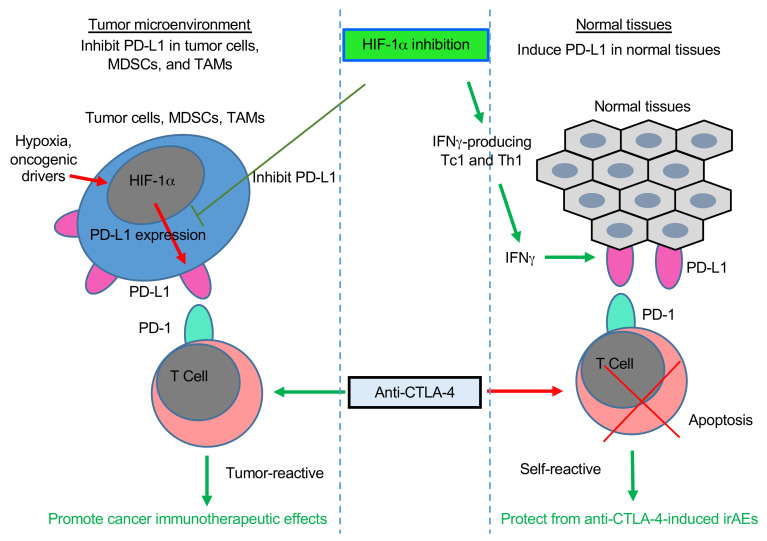

## Introduction

Current immunotherapeutic strategies, articulated as immune checkpoint blockade, aim to release physiological immune tolerance checkpoints for the benefit of immunotherapeutic effect. As such, immune-related adverse events (irAEs) are considered the necessary price for immunotherapy. The relative risk/benefit ratio depends on the significance of the immune checkpoint in immune tolerance versus tumor evasion of host immunity. The PD-1–PD-L1 interaction is less critical than CTLA-4 for immune tolerance, as CTLA-4 inactivation leads to more severe autoimmune diseases than inactivation of PD-1 (1–4). Correspondingly, monoclonal antibodies (mAbs) targeting programmed cell death protein 1 (PD-1) and programmed death ligand 1 (PD-L1) are less toxic than those targeting cytotoxic T lymphocyte antigen 4 (CTLA-4) (5). In terms of therapeutic efficacy, anti–CTLA-4 plus anti–PD-1 combination therapy is considered the most effective immunotherapy strategy (5). However, the combination substantially increases rates of severe irAEs (5) to 50%–90%, depending on therapeutic setting (6–9). Thus, a major challenge for cancer immunotherapy is to eliminate irAEs without compromising synergistic cancer immunotherapeutic effects of dual immune checkpoint blockade.

In the tumor microenvironment (TME), tumor cells and tumor-infiltrating myeloid cells express PD-L1 in response to environmental cues, including cytokines, hypoxia, or growth factors (10–12). PD-L1/B7-H1 causes T cell apoptosis (13) and/or exhaustion upon binding PD-1 (14). Consequently, the PD-1–PD-L1 interaction suppresses T cell–mediated anticancer immunity in the TME, and blocking this interaction reinvigorates immune rejection of tumor cells (15). Although irAEs resulting from anti–PD-1/anti–PD-L1 mAbs are generally less severe than those from anti–CTLA-4 mAbs (16, 17), PD-1/PD-L1 blockade does lead to significant irAEs and administering anti–PD-1 mAbs concurrently with anti–CTLA-4 mAbs substantially worsens irAE incidence and severity (5, 18–21).

A major limitation of anti–PD-1/anti–PD-L1 mAbs is their inability to distinguish PD-1–PD-L1 interactions in the TME, which prevent effective cancer immunity, from PD-1–PD-L1 interactions in normal tissues, which protect against autoimmune diseases. Tumor-specific PD-L1 targeting would be more desirable, as it may achieve cancer immunotherapy without causing irAEs. This may be possible because the molecular mechanisms governing PD-L1 expression in normal tissues and cancer differ. For example, hypoxia, which is one of the major hallmarks distinguishing solid tumors from normal tissues (22), was reportedly responsible for inducing PD-L1 in tumor (23) and myeloid cells (12) via HIF-1α. These findings raised the intriguing possibility that HIF-1α inhibition may selectively repress PD-L1 expression in cancer. Here, we show that pharmaceutical or genetic targeting of HIF-1α suppresses PD-L1 expression in the TME, but paradoxically induces PD-L1 in normal tissues by enhancing T cell production of IFN-γ. Our data demonstrate an approach to differential regulation of PD-L1 for safer and more effective immunotherapy.

## Results

### Targeting HIF-1α suppresses PD-L1 expression in the TME.

**Figure 1:**
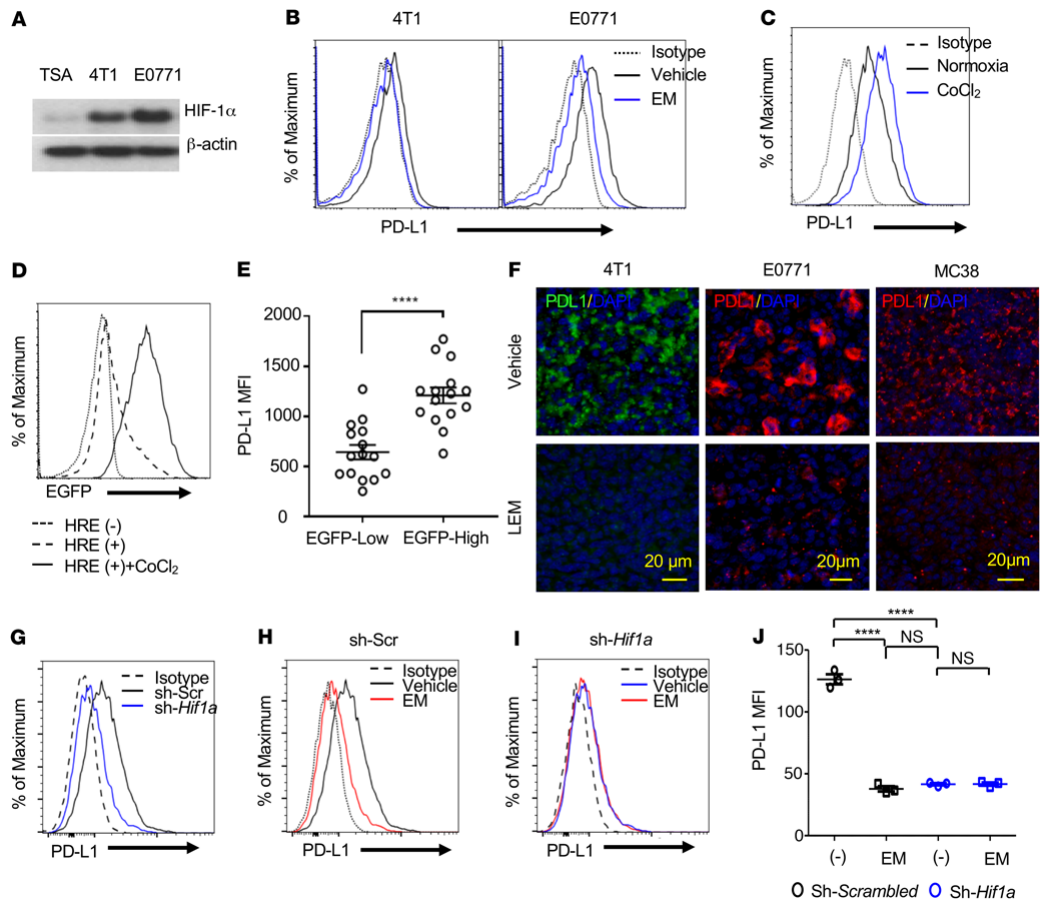
HIF-1α drives PD-L1 expression in tumor cells. (**A**) Western blot of HIF-1α protein in murine breast cancer cells. (**B**) Effect of echinomycin on PD-L1 expression in 4T1 or E0771. Tumor cells were treated with echinomycin (EM, 0.45 nM) or DMSO (vehicle) for 48 hours (1:1000 dilution). Flow cytometry histograms for PD-L1 staining are shown. (**C**) Effect of CoCl_2_ on PD-L1 expression in E0771 cells. E0771 cells were cultured as in **B** with CoCl_2_ (250 μM) or PBS and PD-L1 was measured by flow cytometry. (**D**) 4T1-HRE cells were treated for 24 hours with PBS or CoCl_2_ (250 μM). Flow cytometry histograms for EGFP intensity are shown. (**E**) BALB/c mice received 1 × 10^6^ 4T1-HRE cells (day 0). On day 21, tumors were dissociated and stained for PD-L1. The PD-L1 MFI is plotted for the tumor cells (gated on CD45^–^EGFP^+^ singlets) further divided into top/bottom 30th percentiles based on EGFP. The data are pooled from 3 experiments, presented as mean ± SEM, and were analyzed by Student’s *t* test. (**F**) 4T1, E0771, or MC38 cells were transplanted into BALB/c or C57BL/6 mice, which received vehicle or liposome-encapsulated echinomycin (LEM, 0.25 mg/kg) every other day for a total of 5 doses. Representative PD-L1 immunofluorescence staining is shown for tumor tissues (2 days after final dose). Blue, DAPI. Scale bars: 20 μm. (**G**–**J**) Effects of *Hif1a* shRNA on PD-L1 expression in E0771 cells in vitro. E0771 cells were transduced with lentivirus packaged with scrambled (sh-Scr) or *Hif1a* shRNA (sh-*Hif1a*) and cultured under normoxia for 48 hours with DMSO (–) or echinomycin (EM, 1.35 nM). Flow cytometry histograms of PD-L1 staining are shown, comparing effects of *Hif1a* knockdown (**G**) or effects of echinomycin between sh-Scr (**H**) and sh-*Hif1a* (**I**) cells. (**J**) The data are summarized, expressed as mean ± SEM of PD-L1 MFI for triplicate wells, and were analyzed by 1-way ANOVA with Sidak’s post hoc test. Data are representative of 3 independent experiments. ****P < 0.0001.

Previous studies have shown that hypoxia induces PD-L1 through activation of PD-L1 transcription by HIF-1α. Since tumor cells also express HIF-1α under normoxia, we tested whether the HIF-1α/PD-L1 axis is also active in tumor cells stably expressing HIF-1α under normoxic conditions. We first examined levels of HIF-1α and PD-L1 in the murine breast cancer cell lines 4T1 and E0771 cultured under normoxia. Both cell lines expressed HIF-1α and PD-L1 protein (Figure 1, A and B; see complete unedited blots in the supplemental material), and a reduction in PD-L1 protein was observed in cells treated with the HIF-1α inhibitor echinomycin (Figure 1B). Consistent with previous reports of hypoxia-induced PD-L1 expression (23), treatment of E0771 cells with the hypoxia mimetic CoCl_2_ further upregulated PD-L1 from the basal levels seen in normoxia (Figure 1C). To demonstrate the relationship between HIF-1α activity and PD-L1 protein expression in tumor cells in vivo, we transduced 4T1 cells with a lentiviral transcription factor reporter construct containing the core hypoxia-response element (HRE) motif upstream of an EGFP reporter. In response to CoCl_2_ stimulation, the resultant 4T1-HRE-EGFP cells exhibited a marked increase in EGFP reporter fluorescence (Figure 1D). After engrafting the 4T1-HRE-EGFP cells into immunocompetent BALB/c mice and allowing solid tumors to form, we analyzed PD-L1 expression on the isolated tumor cells by flow cytometry. PD-L1 expression was associated with EGFP reporter activity in the tumor cells (Figure 1E). The results suggest that HIF-1α also regulates PD-L1 expression on tumor cells in vivo. To further test this, we evaluated the effect of liposome-encapsulated echinomycin (LEM) on intratumoral PD-L1 expression by immunofluorescence staining of PD-L1 in the fixed tumor specimens from engrafted tumor cell lines. As shown in Figure 1F, there was a marked reduction in PD-L1 expression in the tumors of LEM-treated mice.

To test whether HIF-1α inhibition is the mechanism responsible for the reduction in PD-L1 protein induced by echinomycin, we used siRNA to knockdown *Hif1a* in E0771 cells and quantified PD-L1 expression by flow cytometry after a 24-hour incubation with vehicle or echinomycin (Figure 1, G–J). Under basal conditions, we found that knockdown of *Hif1a* reduced PD-L1 protein expression (Figure 1, G–J). Moreover, while the inhibitory effect of echinomycin on PD-L1 expression was preserved in E0771 cells transduced with scrambled shRNA (Figure 1, H–J), knockdown of *Hif1a* abrogated the ability of echinomycin to decrease PD-L1 protein (Figure 1, H–J). These results demonstrate that HIF-1α controls PD-L1 expression in E0771 cells and that echinomycin reduced PD-L1 by inhibiting the HIF-1α/PD-L1 axis.

### Immunotherapeutic effect of LEM.

**Figure 2:**
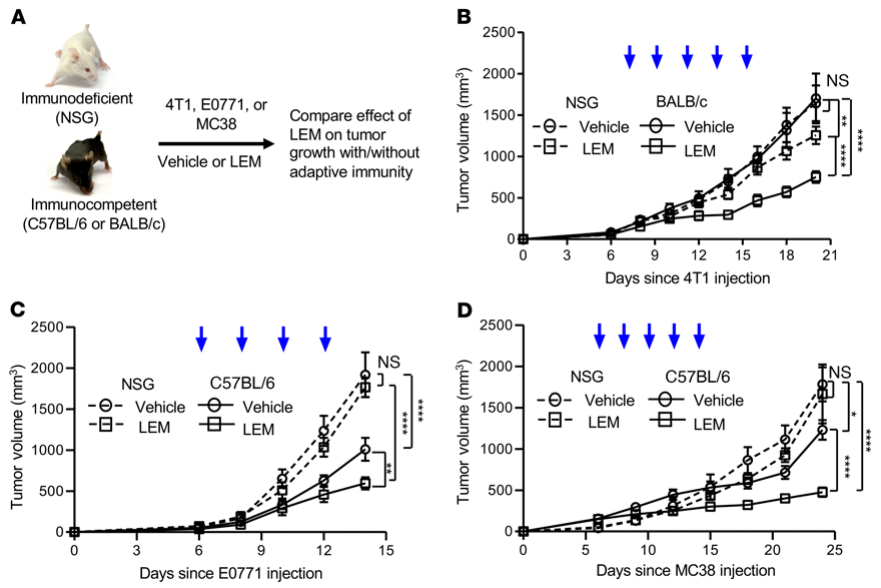
Therapeutic effects of echinomycin on tumor growth in immunodeficient and immunocompetent mice. Three murine tumor lines were tested: 4T1, E0771, and MC38. For each, immunodeficient (NSG) and immunocompetent (BALB/c or C57BL/6) mice were inoculated (day 0), and treatment was initiated with control liposomes (vehicle) or echinomycin liposomes (LEM) on day 6 (blue arrows indicate a single treatment). Tumor growth kinetics were compared to deduce the role of adaptive immunity in the therapeutic effects of echinomycin. (**A**) Diagram of experimental design. (**B**) NSG and BALB/c mice received 4T1 cells (1 × 10^6^/mouse) and were treated with vehicle or 0.15 mg/kg LEM (*n =* 10/group). Mean tumor volumes ± SEM are shown and were analyzed by 2-way ANOVA. The data are representative of 2 independent experiments. (**C**) NSG and C57BL/6 mice received E0771 cells (0.7 × 10^6^/mouse) and were treated with vehicle or 0.25 mg/kg LEM (*n =* 5/group). Mean tumor volumes ± SEM are shown and were analyzed by 2-way ANOVA. The data are representative of 2 independent experiments. (**D**) NSG and C57BL/6 mice received MC38 cells (1 × 10^6^/mouse) and were treated with vehicle or 0.15 mg/kg LEM (*n =* 5/group). Mean tumor volumes ± SEM are shown, which were analyzed by 2-way ANOVA. The data are representative of 2 independent experiments. *P < 0.05; **P < 0.01; ****P < 0.0001.

Given the profound effect of PD-L1 on immune function, it was of interest to test whether HIF-1α inhibition results in an immunotherapeutic effect on cancer. To address this, we first compared the effects of pharmacological HIF-1α inhibition with LEM on tumor growth rate in mice sufficient or deficient in adaptive immunity (Figure 2A). LEM significantly inhibited 4T1 growth in both immunocompetent (BALB/c) and immunodeficient (NSG) recipients compared with each strain’s respective vehicle control (Figure 2B). However, 4T1 growth was more significantly inhibited in immunocompetent mice than in immunodeficient mice, which suggested an immunotherapeutic effect of echinomycin in addition to potentially tumor-intrinsic therapeutic effects in this model. In a second breast cancer model, E0771, the therapeutic effects of LEM were also more pronounced in immune-competent mice (Figure 2C). To test whether HIF-1α inhibition can confer an immunotherapeutic effect in a non–breast cancer model, we repeated the experiments using MC38 murine colon adenocarcinoma cells. As with E0771, all therapeutic effects required immune competence (Figure 2D).

### LEM inhibits PD-L1 in tumor cells by targeting the HIF-1α/PD-L1 axis.

**Figure 3:**
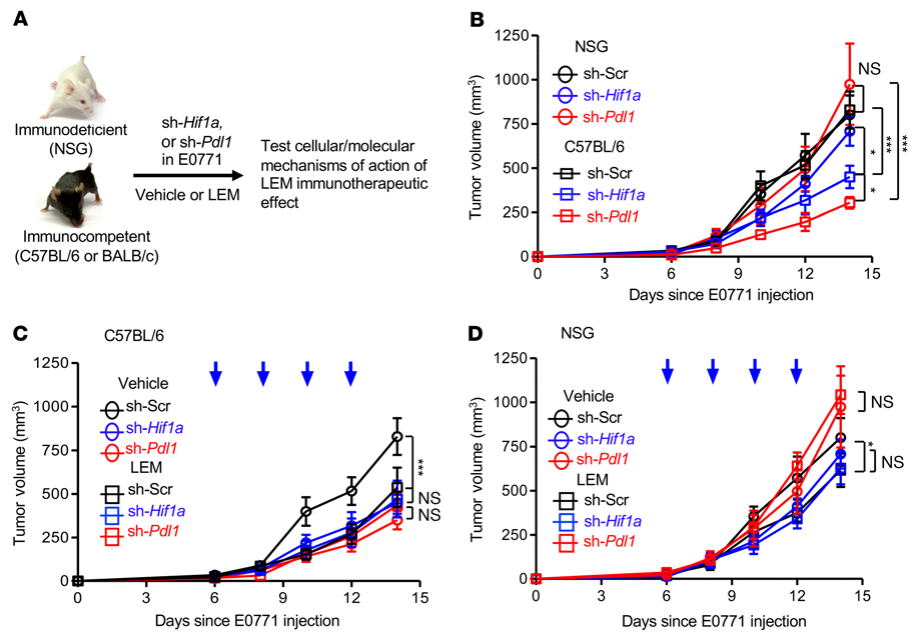
Effects of pharmacological and/or genetic targeting of HIF-1α on E0771 tumor growth in immunodeficient or immunocompetent mice. (**A**) Experimental design. Three sublines of E0771 were generated by lentiviral transduction: scrambled shRNA (sh-Scr), shRNA against *Hif1a* (sh-*Hif1a*), and sh-*Pdl1*. For each, 0.5 × 10^6^ cells were orthotopically transplanted into NSG or C57BL/6 mice (day 0), which received vehicle or echinomycin (LEM, 0.25 mg/kg) starting on day 6. (**B**) Effects of *Hif1a* or *Pdl1* knockdown on E0771 growth among immunodeficient or immunocompetent recipients. (**C**) Effects of vehicle or LEM on sh-Scr, sh-*Hif1a*, or sh-*Pdl1* E0771 growth in immunocompetent recipients. (**D**) Effects of vehicle or LEM on sh-Scr, sh-*Hif1a*, or sh-*Pdl1* E0771 growth in immunodeficient recipients. In the graphs, tumor volumes are plotted as the mean ± SEM for each group (*n =* 5/group), with significance determined by 2-way ANOVA, and the data shown are representative of 2 experiments. *P < 0.05; ***P < 0.001.

Next, we used shRNA to compare the effects of *Hif1a*- or *Pdl1*-targeted knockdown in E0771 cells on the tumor growth kinetics in immunocompetent or immunodeficient recipients. In parallel, we treated both strains with vehicle or LEM to measure the impact of tumor cell–intrinsic *Hif1a* or *Pdl1* on tumor growth in response to LEM treatment (Figure 3A). In C57BL/6, but not NSG recipients, genetic depletion of *Hif1a* (sh-*Hif1a*) in E0771 cells significantly inhibited tumor growth compared with E0771 transduced with scrambled shRNA (sh-Scr) in mice of the same respective strains (Figure 3B). Moreover, the tumor growth rates of E0771 with *Hif1a* knockdown were also significantly reduced in immunocompetent versus immunodeficient recipients (Figure 3B). As in Figure 2C, LEM more effectively inhibited sh-Scr E0771 tumor growth in C57BL/6 (Figure 3C) compared with NSG (Figure 3D) recipients; in contrast, LEM did not inhibit sh-*Hif1a* E0771 tumor growth, regardless of the recipient strain (Figure 3, C and D). Thus, pharmacologic or genetic targeting of HIF-1α in tumor cells alone can confer an immunotherapeutic effect. Furthermore, the loss of biological activity of echinomycin following knockdown of *Hif1a* in E0771 provides genetic evidence that echinomycin confers an immunotherapeutic effect in vivo by targeting the HIF-1α in tumor cells.

In the same manner, we analyzed the effects of *Pdl1* knockdown to determine whether downregulation of PD-L1 is critical in the immunotherapeutic effect of echinomycin. Much like the knockdown of *Hif1a*, loss of *Pdl1* (sh-*Pdl1*) in E0771 cells also inhibited tumor growth in C57BL/6, but not NSG, recipients (Figure 3B), and LEM did not further suppress sh-*Pdl1* E0771 tumor growth in C57BL/6 (Figure 3C) or NSG (Figure 3D) recipients. Taken together, the data support the conclusion that echinomycin confers immunotherapeutic effects in vivo by targeting the HIF-1α/PD-L1 axis in tumor cells.

### HIF-1α inhibition potentiates anti–CTLA-4 immunotherapy.

**Figure 4:**
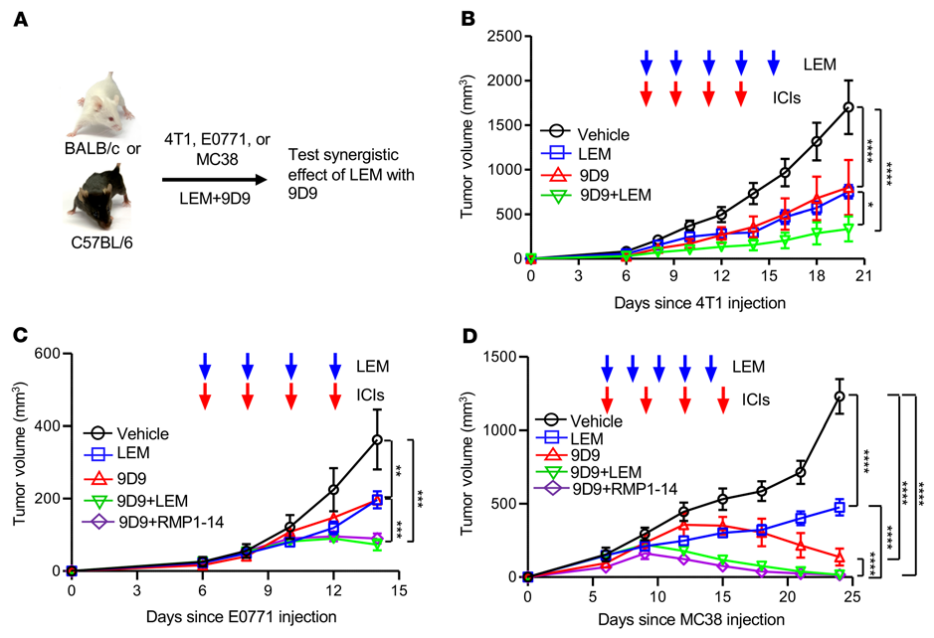
Echinomycin potentiates therapeutic effect of anti–CTLA-4 antibody. In 4T1, E0771, or MC38 syngeneic tumor models, the effects of echinomycin (LEM) plus anti–CTLA-4 (9D9) on tumor growth were tested in comparison to either monotherapy or vehicle control. In E0771 and MC38 models, effect of 9D9 plus anti–PD-1 (RMP1-14) was also assessed. Treatment was initiated on day 6 after tumor cell inoculation, with single treatments indicated by the blue (LEM) and red arrows (mAbs). In the graphs, mean tumor volumes ± SEM are shown for each group and were analyzed by 2-way ANOVA. (**A**) Diagram of experimental design. (**B**) Effects of 9D9 plus LEM on syngeneic 4T1 tumor growth. BALB/c mice with 4T1 tumors received vehicle, LEM (0.15 mg/kg/dose), 9D9 (0.2 mg/mouse/dose), or combination (*n =* 10/group). Data shown for 1 of 3 independent experiments. ICIs, immune checkpoint inhibitors (mAbs). (**C**) Effects of 9D9 plus LEM on syngeneic E0771 growth. E0771 cells (0.5 × 10^6^) were orthotopically transplanted into C57BL/6 mice, which received vehicle, LEM (0.25 mg/kg), and/or various mAbs (0.2 mg/mouse/dose) (*n =* 5/group). Representative data shown for 1 of 3 independent experiments. (**D**) Effects of 9D9 plus LEM on syngeneic MC38 growth. MC38 cells (1 × 10^6^) were transplanted into the left flank of C57BL/6 mice. Mice received vehicle, LEM (0.15 mg/kg), and/or various mAbs (0.2 mg/mouse/dose) (*n =* 5/group). Representative data shown for 1 of 3 independent experiments. *P < 0.05; **P < 0.01; ***P < 0.001; ****P < 0.0001.

Cotargeting CTLA-4 and PD-1/PD-L1 immune checkpoints simultaneously with their respective blocking mAbs is the most efficacious strategy currently available for cancer immunotherapy. Having established that LEM can target PD-L1 in tumor cells and promote an immunotherapeutic effect in vivo, we next asked whether this strategy may potentiate immunotherapeutic effects in the context of anti–CTLA-4 therapy. We examined the therapeutic effects of CTLA-4–blocking mAbs, with or without LEM, using 4T1, E0771, or MC38 syngeneic mouse models of cancer (Figure 4A). As shown in Figure 4B, anti–mouse CTLA-4 mAb (9D9) in combination with LEM significantly inhibited 4T1 tumor growth more effectively than either monotherapy alone. To further investigate the combined efficacy of targeting HIF-1α during anti–CTLA-4 therapy, we performed similar drug-treatment experiments using immunocompetent C57BL/6 recipients and the E0771 breast cancer (Figure 4C) or MC38 colon adenocarcinoma (Figure 4D) model and observed synergistic effects in all models. We also compared the effects of LEM or anti–PD-1 (RMP1-14) in conjunction with 9D9. Again, we observed significant inhibition of tumor growth by LEM or 9D9 monotherapy compared with vehicle, while the greatest inhibition was achieved by 9D9 plus LEM or RMP1-14 (Figure 4, C and D). These data demonstrated that the therapeutic effect of blocking the PD-1–PD-L1 interaction can be similarly achieved by either anti–PD-1 or echinomycin.

### LEM inhibits PD-L1 on tumor cells and tumor-infiltrating myeloid cells.

**Figure 5:**
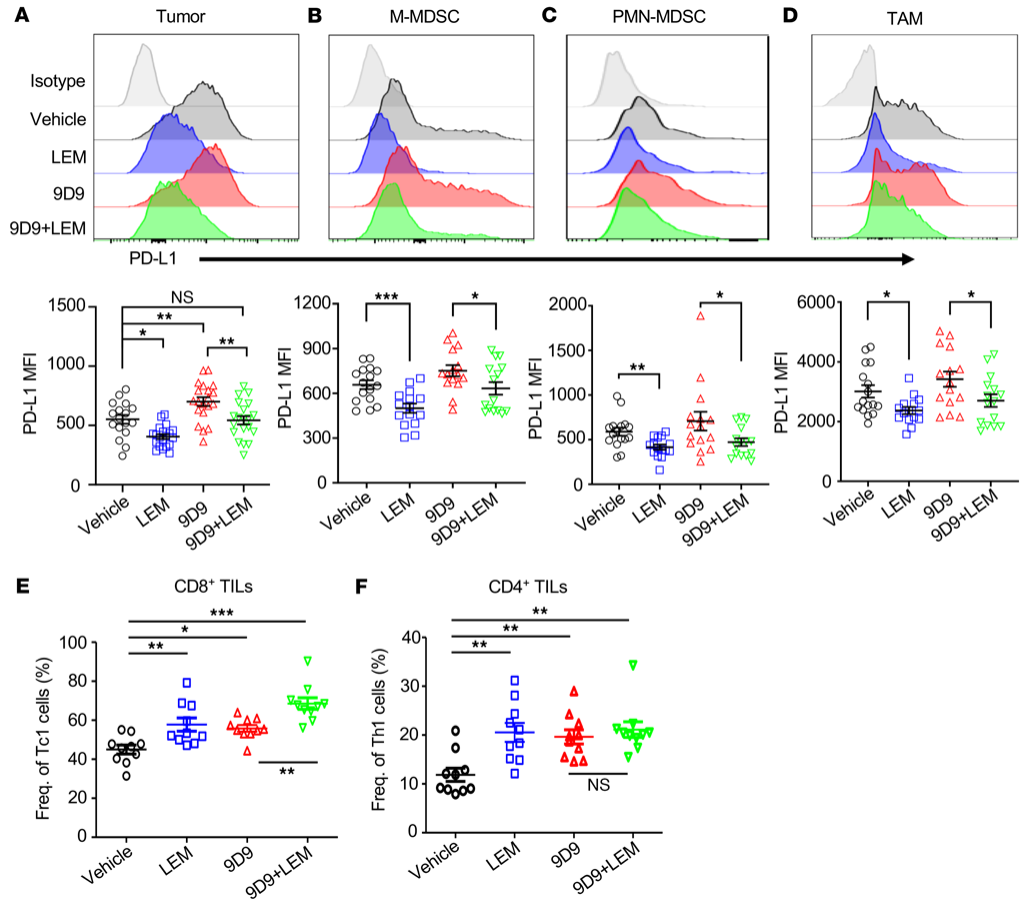
Echinomycin suppresses PD-L1 on tumor cells and tumor-infiltrating myeloid cells and expands the IFN-γ–producing CD8^+^ and CD4^+^ T cells with or without anti–CTLA-4 antibodies. C57BL/6 mice received E0771 cells (0.5 × 10^6^/mouse) on day 0 followed by treatment with vehicle, echinomycin (LEM, 0.25 mg/kg/dose), anti–CTLA-4 (9D9, 0.2 mg/mouse/dose), or 9D9 plus LEM on days 6, 8, 10, and 12. On day 14, the tumors were analyzed by flow cytometry. (**A**–**D**) PD-L1 expression on tumor and tumor-associated myeloid cells. PD-L1 expression was analyzed on tumor cells (gated on live CD45^–^ singlets) (**A**), M-MDSCs (gated on live CD45^+^CD11b^+^CD11c^–^Ly6C^hi^Ly6G^–^ singlets) (**B**), PMN-MDSCs (gated on live CD45^+^CD11b^+^CD11c^–^Ly6C^int^Ly6G^+^ singlets) (**C**), or CD11c^+^ TAMs (gated on live CD45^+^CD11b^+^CD11c^+^ singlets) (**D**). Upper panels show representative histograms of PD-L1. In the lower panels, dot plots show the PD-L1 MFI for individual mice from 3 independent experiments (*n =* 5 mice/group/experiment). The data are presented as the mean ± SEM of PD-L1 MFI and were analyzed by 1-way ANOVA with Sidak’s multiple-comparison test (**A**) or by 2-tailed, unpaired Student’s *t* test (**B**–**D**). (**E** and **F**) Frequency of TILs producing IFN-γ. The frequencies of CD8^+^IFN-γ^+^ (Tc1) among total CD8^+^ TILs (**E**) and CD4^+^IFN-γ^+^ (Th1) among total CD4^+^ TILs (**F**) are shown. The tumor cell suspensions were cultured for 4 hours in the presence of PMA plus ionomycin and GolgiStop prior to staining. The dot plots show the Tc1 or Th1 cell frequencies for individual mice from 2 independent experiments (*n =* 5 mice/group/experiment), which were analyzed by 1-way ANOVA with Sidak’s multiple-comparison test. *P < 0.05; **P < 0.01; ***P < 0.001.

We have shown that systemic HIF-1α inhibition suppressed PD-L1 expression in multiple tumors (Figure 1F). To gain insight into the cellular landscape and cell-specific expression patterns of PD-L1 in the TME following LEM and/or 9D9 treatment, we analyzed E0771 tumors from C57BL/6 mice treated with vehicle, LEM, 9D9, or 9D9 plus LEM for the composition of immune cells. While LEM did not significantly impact the frequencies of tumor-infiltrating lymphocyte (TIL) or tumor-infiltrating myeloid subsets, 9D9 reduced the frequencies of polymorphonuclear myeloid-derived suppressor cells (PMN-MDSCs) (Supplemental Figure 1; supplemental material available online with this article; https://doi.org/10.1172/JCI150846DS1). However, LEM significantly reduced PD-L1 expression on tumor cells (Figure 5A), and tumor-infiltrating monocytic MDSCs (M-MDSCs) (Figure 5B), PMN-MDSCs (Figure 5C), and CD11b^+^CD11c^+^ double-positive cells (Figure 5D), with or without anti–CTLA-4 therapy. Further analysis revealed that most of the CD11b^+^CD11c^+^ cells were tumor-associated macrophages (TAMs), as roughly 90% expressed F4/80, consistent with a previous report (ref. 24 and Supplemental Figure 2). The results show that, in addition to tumor cells, in vivo HIF-1α inhibition can also suppress PD-L1 on tumor-infiltrating myeloid cells, and these effects persist in the context of anti–CTLA-4 therapy. More importantly, the results provide evidence that HIF-1α is involved in coordinating PD-L1 expression on tumor-infiltrating myeloid cells in the TME.

To test whether HIF-1α inhibition can rescue TIL function in the TME, we used flow cytometry to measure frequencies of IFN-γ–expressing T cells in the E0771 tumors. Compared with vehicle, all treatments increased the frequencies of both IFN-γ^+^CD8^+^ (Tc1) and IFN-γ^+^CD4^+^ (Th1) subsets, although the highest frequencies of Tc1 were observed in mice receiving 9D9 plus LEM (Figure 5, E and F). The absolute numbers of Tc1 and Th1 cells were also highest in 9D9 plus LEM–treated mice, as shown in Supplemental Figure 3.

Apart from boosting CD8^+^ TIL responses, anti–PD-L1 promoted an inflammatory TAM phenotype in the TME, which may contribute to its cancer immunotherapeutic effect (25). In this regard, we also noted increased MHCII expression in 9D9 plus LEM–treated versus vehicle-treated mice (Supplemental Figure 4).

**Figure 6:**
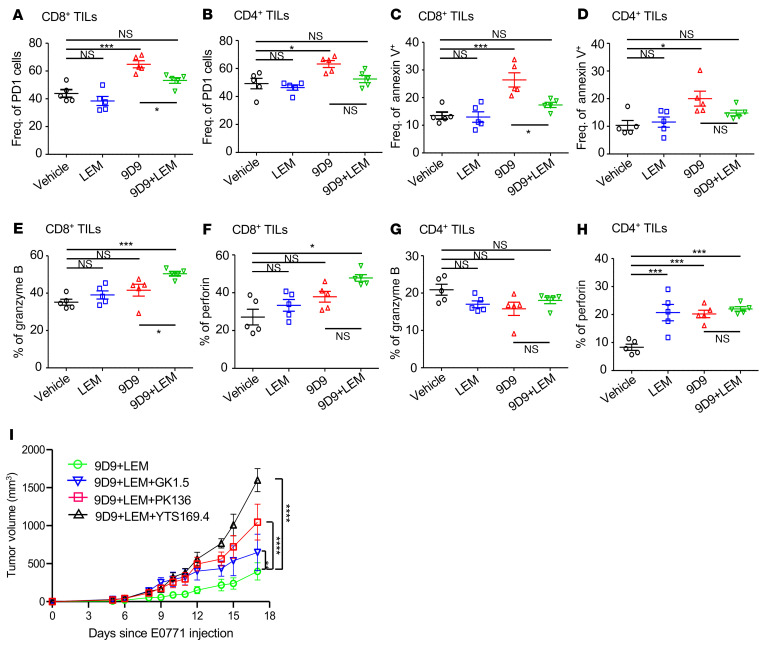
Echinomycin improves TIL function in anti–CTLA-4–treated mice and CD8^+^ TILs are critical for combination efficacy. (**A**–**H**) C57BL/6 mice received E0771 cells (0.5 × 10^6^/mouse) on day 0 followed by treatment with vehicle, echinomycin (LEM, 0.25 mg/kg/dose), 9D9 (0.2 mg/mouse/dose), or 9D9 plus LEM on days 6, 8, and 10. On day 14, tumors were analyzed by flow cytometry. Each graph shows the frequencies of CD8^+^ or CD4^+^ subsets among TILs (gated on live CD45^+^CD3^+^CD8^+^CD4^–^ singlets or live CD45^+^CD3^+^CD8^–^CD4^+^ singlets). Data are representative of 2 experiments and presented as the mean ± SEM of each group (*n =* 5/group), and were analyzed by unpaired, 2-tailed Student’s *t* test. (**A** and **B**) Frequencies of TILs expressing PD-1. (**C** and **D**) Frequencies of annexin V^+^ TILs. (**E** and **F**) Frequencies of CD8^+^ TILs expressing granzyme B or perforin. (**G** and **H**) Granzyme B and perforin expression in CD8^+^ TILs. (**I** and **J**) Granzyme B and perforin expression in CD4^+^ TILs. (**J**) Effect of depletion of CD4^+^, CD8^+^, or NK cells on tumor growth inhibition by 9D9 plus LEM in the syngeneic E0771 model. C57BL/6 mice received E0771 cells (0.5 × 10^6^/mouse) on day 0. On day 5, the mice were randomized to receive depleting antibodies (500 μg of anti-CD4 [GK1.5], anti-CD8 [YTS169.4], anti-NK1.1 [PK136], or isotype control). All groups received 9D9 (200 μg) on day 6, and LEM (250 μg/kg) on days 6, 8, and 10. Mice received supplemental dose of depleting antibodies (200 μg) on days 8 and 10. The mean ± SEM tumor volumes are plotted on the *y* axes for each group (*n =* 5/group) and were analyzed by 2-way ANOVA. Representative data shown for 1 of 2 experiments. *P < 0.05; ***P < 0.001; ****P < 0.0001.

To better understand the impact of pharmacologic HIF-1α targeting in the context of immunotherapy, we performed more detailed analysis of TILs. E0771 mice treated with 9D9 had higher expression of exhaustion marker PD-1 on CD8^+^ TILs compared with vehicle, which was reversed by LEM (Figure 6A). The same was seen for PD-1 expression on CD4^+^ TILs (Figure 6B), and to a lesser extent, for CTLA-4 expression on CD8^+^ TILs (Supplemental Figure 5A). The drug treatments had minimal impact on CD4^+^ TIL expression of CTLA-4 (Supplemental Figure 5B). In addition to TIL exhaustion (14), PD-L1 can also induce TIL apoptosis (13). Annexin V staining revealed more apoptotic CD8^+^ and CD4^+^ TILs in tumors of 9D9-treated mice compared with vehicle, and adding LEM appeared to repress this effect in CD8^+^ TILs (Figure 6C). The same trend was seen for CD4^+^ TILs, although the difference between the 9D9 and 9D9 plus LEM groups was not significant (Figure 6D). Higher expression of cytolytic effector molecules granzyme B and perforin were noted in CD8^+^ TILs of 9D9 plus LEM–treated mice versus vehicle (Figure 6, E and F). Roughly one-fifth of CD4^+^ TILs were granzyme B^+^, which was not significantly affected by drug treatments (Figure 6G). On the other hand, in all treated groups, the mean frequencies of CD4^+^ TILs expressing perforin were roughly double that of the control group (Figure 6H).

We used depleting antibodies to assess the impact of CD4^+^, CD8^+^, and NK cells on the combined efficacy of 9D9 plus LEM in E0771 mice. These studies revealed that optimal efficacy required all 3 cell types, with CD8^+^ cells being the most critical, followed by NK and CD4^+^ cells (Figure 6I). Thus, the immunotherapeutic effects of pharmacological HIF-1α inhibition in the context of anti–CTLA-4 are multiple-cell dependent, but primarily depend on CD8^+^ T cells.

Since HIF-1α regulates Treg and Th differentiation (26), we further examined the impact of LEM on lineage-specific transcription factors and cytokines in the TILs. The proportions of CD8^+^ and CD4^+^ TILs expressing T-bet were significantly increased in 9D9 plus LEM–treated mice versus vehicle (Supplemental Figure 5, C and D). TNF-α was unaffected by the therapies in CD4^+^ TILs, although we observed increased TNF-α^+^CD8^+^ TILs in groups receiving 9D9 (Supplemental Figure 5, E and F). The proportions of CD8^+^ and CD4 ^+^TILs expressing RORγt were unchanged among different groups (Supplemental Figure 5, G and H). None of the therapies significantly impacted frequencies of tumor-infiltrating Treg or Th17 cells, except for a modest decrease in Tregs for groups receiving 9D9 (Supplemental Figure 5, I and J). The proportion of tumor-infiltrating NK cells expressing granzyme B and perforin were decreased and increased, respectively, in mice treated with 9D9 alone (Supplemental Figure 5, K and L). 9D9 significantly increased and decreased the frequencies of effector and memory CD8^+^ T cells in the tumors, respectively (Supplemental Figure 6).

LEM alone inhibited PD-L1 on tumor cells and tumor-infiltrating myeloid cells but increased the proportion of CD8^+^ TILs expressing IFN-γ (Figure 5). An important question arose as to whether echinomycin improves CD8^+^ TIL function directly by a T cell–intrinsic mechanism, or indirectly through reducing PD-L1 on tumor and/or myeloid cells. To test this, we generated mice with conditional knockout of *Hif1a* in T lineages using the Cre-lox system. Loss of *Hif1a* did not significantly impact the proportion of CD4^+^ and CD8^+^ TILs expressing IFN-γ, T-bet, or RORγt, or the frequencies of Tregs or Th17 cells. In CD4^+^, but not CD8^+^ TILs, we found an increased frequency of PD-1^+^ cells (Supplemental Figure 7). Granzyme B and perforin in CD8^+^ TILs was slightly reduced in *Hif1a*-KO mice, but not significantly. In contrast, knockdown of *Pdl1* in the tumor cells significantly increased the frequencies of Tc1 and Th1 cells, phenocopying the effects of LEM (Supplemental Figure 8). Therefore, suppression of PD-L1 on tumor cells can at least partially account for the enhanced CD8^+^ TIL function and therapeutic effects provided by LEM in the immune-competent mouse. Notably, inhibition of PD-L1 on tumor cells by LEM was preserved in mice with conditional knockout of *Hif1a* in T cells. These data indicate that the decreased PD-L1 expression is not due to a T cell–intrinsic effect of echinomycin (Supplemental Figure 9).

Sitkovsky’s group previously reported that TILs tend to avoid hypoxic zones in the TME (27). Using methods from Hatfield et al. (27), we noted an increase in CD3^+^ TIL infiltration into hypoxic areas of the tumors in LEM-treated mice (Supplemental Figure 10).

### LEM induces PD-L1 expression to limit anti–CTLA-4–induced T cell infiltration in irAE target organs.

**Figure 7:**
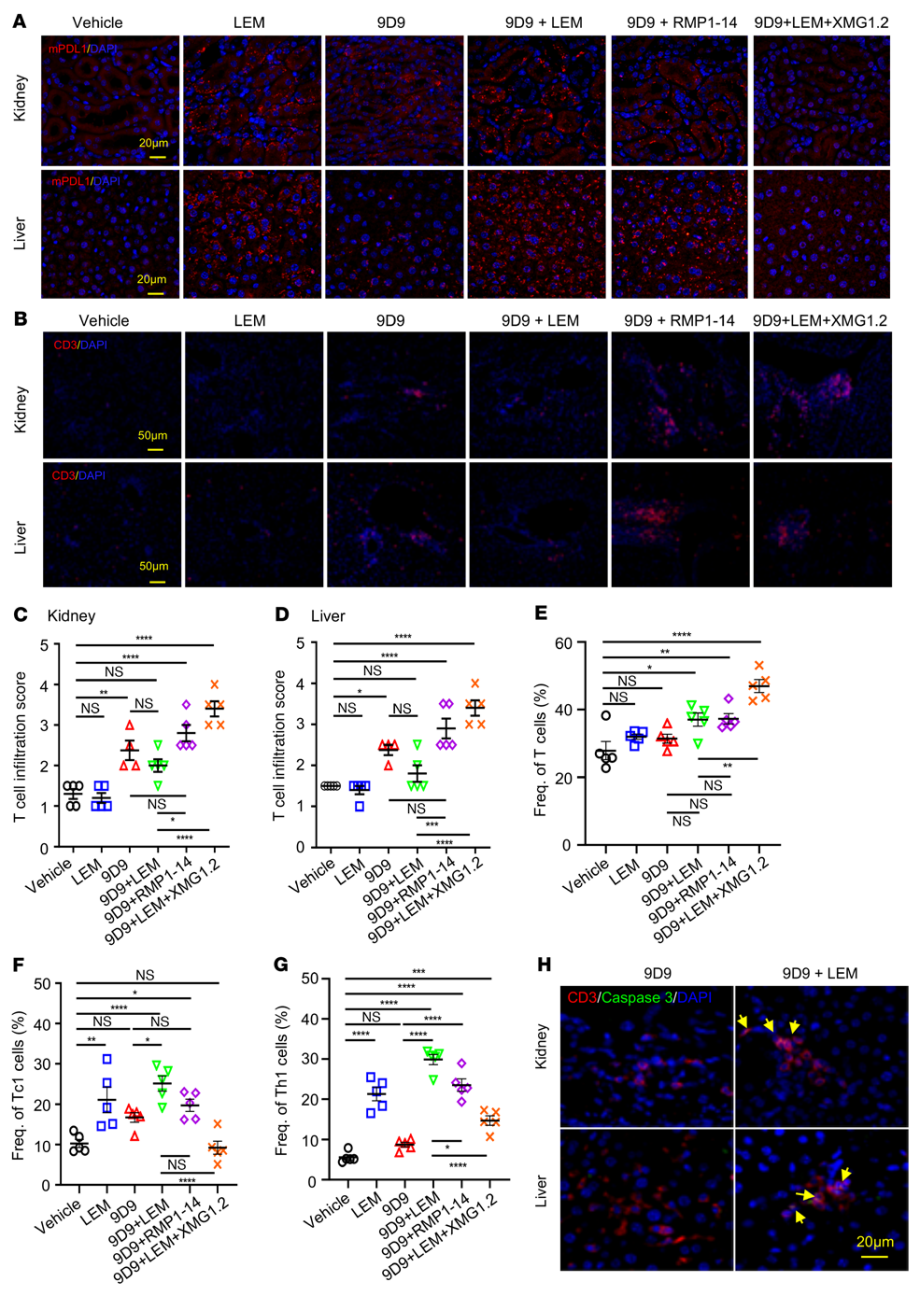
Echinomycin stimulates PD-L1 expression in irAE target organs to limit the infiltration of T cells caused by anti–CTLA-4 mAbs by an IFN-γ–dependent mechanism. E0771 cells (0.5 × 10^6^) were transplanted into C57BL/6 mice (day 0), which were divided into 6 treatment groups (*n =* 5/group): vehicle, echinomycin (LEM), anti–CTLA-4 (9D9), 9D9 plus LEM, 9D9 plus LEM plus anti–IFN-γ (XMG1.2), and 9D9 plus anti–PD-1 (RMP1-14). LEM (0.25 mg/kg) or mAbs (0.2 mg/mouse/dose) were given on days 6, 8, 10, and 12. On day 14, the mice were perfused. Dissociated spleens were stimulated for 4 hours with PMA plus ionomycin and GolgiStop prior to flow cytometry. Liver and kidney tissues were fixed and immunofluorescently stained for indicated markers and with DAPI (blue). (**A**) PD-L1 expression in the tumor-bearing mice treated with different therapies. Representative PD-L1 immunofluorescence staining shown for kidney and liver tissues from indicated treatment groups. Scale bars: 20 μm. (**B**–**D**) T cell infiltration in the liver and kidney of tumor-bearing mice. (**B**) Representative CD3 immunofluorescence staining depicting T cell infiltration in kidney and liver tissues. Scale bars: 50 μm. (**C** and **D**) T cell infiltration was scored (scale of 0–4) in the kidney (**C**) and liver (**D**) tissues as follows: 0, normal/none; 1, minimal; 2, mild; 3, moderate; 4, severe. (**E**) Frequency of splenic T cells (gated on live CD45^+^CD3^+^CD8^+^CD4^–^ singlets) among total hematopoietic cells (gated on live CD45^+^ singlets). (**F**) Frequency of splenic Tc1 cells among CD8^+^ T cells (gated on live CD45^+^CD3^+^CD8^+^CD4^–^ singlets). (**G**) Frequency of splenic Th1 cells among CD4^+^ T cells (gated on live CD45^+^CD3^+^CD8^–^CD4^+^ singlets). (**H**) CD3 and cleaved caspase 3 staining in kidney and liver. Representative immunofluorescence images shown for kidney (upper) and liver tissues (lower) of mice that received 9D9 or 9D9 plus LEM. All data are representative of at least 2 independent experiments. Scale bar: 20 μm. In **C**–**G**, data are presented as the mean ± SEM, with each dot representing an individual mouse, and were analyzed by 1-way ANOVA with Sidak’s post hoc test. *P < 0.05; **P < 0.01; ***P < 0.001; ****P < 0.0001.

To test whether PD-L1 is induced at the tissue level in response to anti–CTLA-4 therapy, we performed immunofluorescence staining of PD-L1 and CD3 in the liver and kidney of tumor-bearing mice treated with 9D9 alone or in combination with other therapies (Figure 7A). PD-L1 expression in these tissues was elevated in mice treated with 9D9 (Figure 7A). Interestingly, LEM also induced PD-L1 (Figure 7A), but only 9D9 resulted in hepatic and renal infiltration of T cells (Figure 7, B–D). T cell infiltration was reduced in 9D9 plus LEM–treated mice when compared with 9D9 alone (Figure 7, B–D). In contrast, the frequency of T cells as well as those of Tc1 and Th1 expanded in the spleen of mice that received 9D9 plus LEM treatment (Figure 7, E–G). Thus, the reductions in T cells in liver and kidney induced by LEM were not the result of general T cell inactivation. Rather, cleaved caspase 3 staining suggested that the induced PD-L1 regulates T infiltration by triggering apoptosis (Figure 7H). When 9D9 was combined with anti–PD-1 (RMP1-14), the mice had high T cell infiltration in liver and kidney (Figure 7, B–D). The frequencies of T cells in the spleen, including Tc1 and Th1, were comparable when either anti–PD-1 or LEM was used in conjunction with anti–CTLA-4 mAb (Figure 7, E–G). Since IFN-γ is known to upregulate PD-L1 in normal tissues (28, 29), we hypothesized that PD-L1 induction by LEM could be due to IFN-γ. LEM alone did not stimulate increased infiltration of T or NK cells in the tissues with PD-L1 expression (Figure 7, B–D, and Supplemental Figure 11), but led to a modest increase in IFN-γ detected in the serum (Supplemental Figure 12).

We further tested the importance of IFN-γ by using the anti–IFN-γ neutralizing mAb XMG1.2, which abrogated PD-L1 induction by 9D9 plus LEM treatment and increased T cell infiltration in the kidneys and liver (Figure 7, B–D). XMG1.2 also abrogated PD-L1 expression in the kidney and liver in the absence of 9D9, indicating that IFN-γ is responsible for PD-L1 induction by LEM in these tissues (Supplemental Figure 13). Conditional knockout of *Hif1a* in T cells did not phenocopy the effects of LEM on PD-L1 induction in the liver, but PD-L1 induction was preserved regardless of the mouse genotype (Supplemental Figure 14). To test whether LEM can reduce irAEs in the adult tumor-bearing mouse, we measured serum biomarkers for hepatic, renal, and gastrointestinal (GI) irAEs. However, the adult mouse tolerated a high dose of anti–CTLA-4 antibody without significant irAEs (Supplemental Figure 15).

### LEM protected ipilimumab-induced irAEs in human CTLA4–knockin mice.

**Figure 8:**
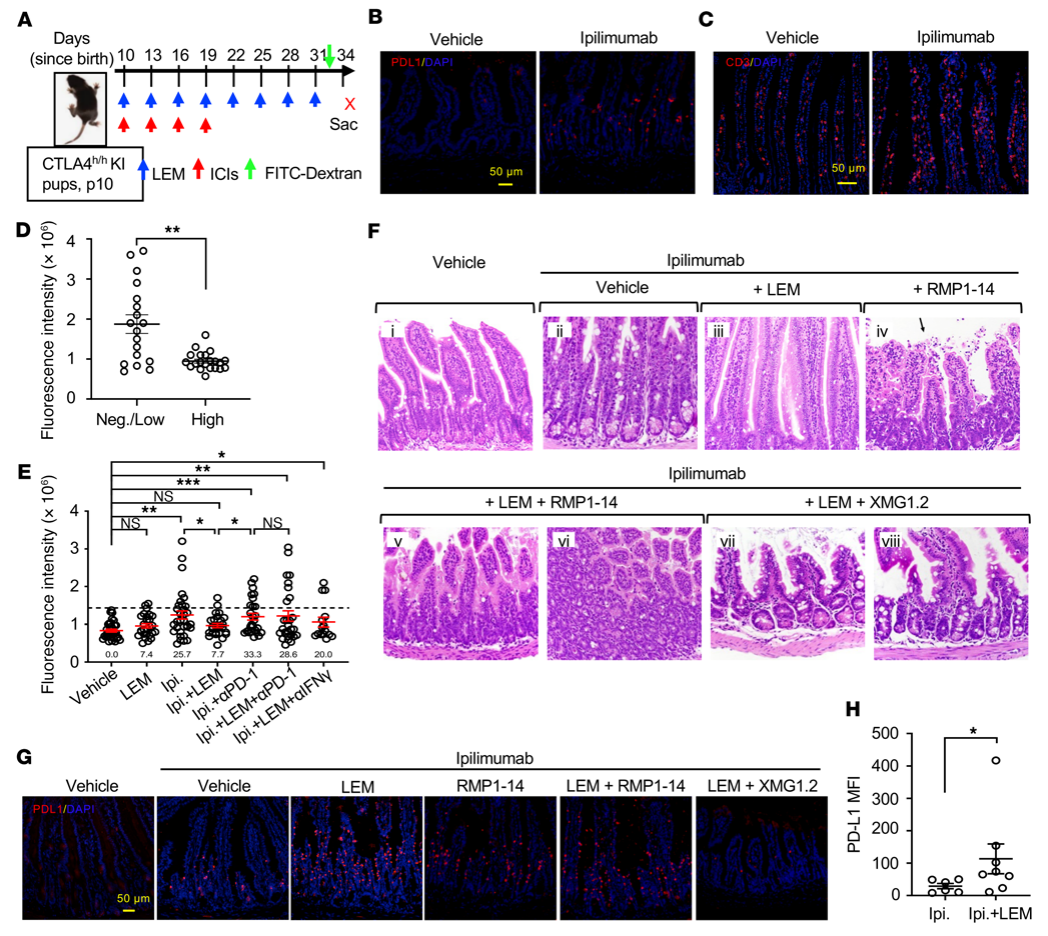
Echinomycin induces PD-L1 to counter ipilimumab-induced GI irAEs by an IFN-γ–dependent mechanism. (**A**) Experimental design. Ipilimumab (Ipi.) was used to induce GI irAEs in *CTLA4*^h/h^ pups (detailed in Methods). Single dose of ipilimumab and other agents are indicated in the diagram by arrows (red, mAbs; blue, vehicle/LEM). ICIs, immune checkpoint inhibitors (mAbs). (**B** and **C**) Representative immunofluorescence images showing T cell infiltration (**B**) or PD-L1 expression (**C**) in jejunum of vehicle- or ipilimumab-treated mice. (**D**) Association of intestinal PD-L1 expression with GI irAEs determined by FITC-dextran assay. Intestinal PD-L1 expression was scored as negative/low (*n =* 18) or high (*n =* 21) based on immunofluorescence, and serum FITC-dextran intensity is presented as the mean ± SEM for each group, analyzed by Student’s *t* test. Aggregate data shown from 4 experiments. (**E**–**G**) Effects of LEM, anti–PD-1 (RMP1-14), and anti–IFN-γ (XMG1.2) in the GI irAE model. Mice were grouped as follows to receive therapies based on the experimental design depicted in **A**: vehicle (*n =* 33), LEM (*n =* 27), ipilimumab (*n =* 33), ipilimumab plus LEM (*n =* 26), ipilimumab plus RMP1-14 (*n =* 27), ipilimumab plus LEM plus RMP1-14 (*n =* 28), and ipilimumab plus LEM plus XMG1.2 (*n =* 15). (**E**) Serum FITC-dextran intensity shown as mean ± SEM for individual mice pooled from 3 independent experiments. GI irAE incidence corresponding to each group is annotated (percentages); dotted line represents the threshold for the determination of GI irAEs. Statistics were determined by 2-tailed, unpaired Student’s *t* test. (**F**) Representative H&E images from intestines of mice receiving different therapies. Panel iv shows cellular debris and necrosis in lamina propria and epithelium (arrow). (**G**) Representative immunofluorescence images showing PD-L1 staining in jejunum of mice from different treatment groups. Scale bars: 50 μm (**B**, **C**, and **G**). (**H**) Flow cytometry analysis of PD-L1 expression in intestinal epithelial cells (gated on live CD45^–^cytokeratin^+^ singlets) from mice treated with ipilimumab (*n =* 6) or ipilimumab plus LEM (*n =* 8). Data shown as mean ± SEM of the PD-L1 MFI for each mouse, and were analyzed by 2-tailed, unpaired Student’s *t* test. *P < 0.05; **P < 0.01; ***P < 0.001.

To circumvent this caveat, we used human *CTLA4*–knockin (*CTLA4*^h/h^-KI) mice, which are susceptible to irAE induction by ipilimumab at a young age (30). The GI tract is the most frequent target of irAEs (31). Therefore, we used intestinal permeability to orally administered FITC-dextran and histology as the readout for irAEs (Figure 8A). Similar to what was described for liver and kidney, ipilimumab treatment resulted in elevated PD-L1 expression (Figure 8B) and T cell accumulation (Figure 8C) in the intestines. To explore whether PD-L1 could serve a functional role in the protection from GI irAEs induced by ipilimumab, we compared the fluorescence intensity of FITC-dextran measured in the sera among those with high or low PD-L1 staining in the intestines. We observed that mice with high levels of intestinal PD-L1 had much lower intestinal permeability (Figure 8D). The association between intestinal permeability and PD-L1 expression supports the hypothesis that ipilimumab-induced PD-L1 serves as a limiting factor against ipilimumab-induced GI irAEs.

To test this hypothesis, we assessed whether blockade of the PD-1–PD-L1 checkpoint during ipilimumab treatment would also worsen GI irAEs in the *CTLA4*^h/h^-KI mouse model, and how this approach might compare to substitution of anti–PD-1 mAbs with LEM. We evaluated the percentage of mice with significantly higher serum FITC-dextran than control mice, using the mean plus 2 standard deviations as a boundary for intestinal leakage. LEM protected against ipilimumab-induced intestinal leakage by a PD-L1–dependent mechanism, as this protection was abrogated by anti–PD-1 (Figure 8E). Moreover, in mice that received ipilimumab plus LEM treatment, the addition of anti–IFN-γ mAb increased the frequency of mice with intestinal leakage from 7.7% to 20.0% (Figure 8E). Collectively, the data suggested that through induction of IFN-γ, echinomycin confers protection against ipilimumab-induced GI irAEs by elevating PD-L1 expression to fortify the PD-1–PD-L1 checkpoint.

To further investigate PD-L1 expression in the intestinal tissues in response to ipilimumab and to validate its role in conferring protection from ipilimumab-induced GI irAEs, we performed histological analysis of the intestinal tissue and immunofluorescence staining of PD-L1. Consistent with the FITC-dextran data in Figure 8E, ipilimumab induced intestinal inflammation (see Supplemental Figure 16 for additional information). The inflammation was largely abrogated by LEM, as mice treated with ipilimumab plus LEM for the most part exhibited normal intestinal pathology (Figure 8F). LEM enhanced PD-L1 expression in the intestine compared with ipilimumab alone (Figure 8G). The elevated PD-L1 expression was confirmed by flow cytometry using digested intestinal tissues (Figure 8H).

To confirm the significance of induced PD-L1 in protection against inflammation in the intestine, we used an anti–PD-1 mAb to block the PD-1–PD-L1 interaction. These data showed that the protective effect of LEM is abrogated by the anti–PD-1 mAb (Figure 8F). Moreover, IFN-γ was increased in intestines from mice that received treatment with both LEM and ipilimumab (Supplemental Figure 17), and the protective effect of LEM depends on IFN-γ because the effect was abolished by the anti–IFN-γ mAb XMG1.2 (Figure 8F). These data suggested that the IFN-γ/PD-L1 axis is responsible for the echinomycin-mediated protection against ipilimumab-induced GI irAEs.

## Discussion

HIF-1α inhibition is an area of active investigation in cancer therapy (32, 33). We have reported that echinomycin effectively eliminated leukemia stem cells (34). However, clinical development of echinomycin for solid tumors has met with minimal success. In our studies of breast cancer, we found that reformulating echinomycin with liposomes (LEM) enabled potent therapeutic effects in orthotopic xenograft mouse models of triple-negative breast cancer, including primary tumor growth and metastasis in the MDA-MB-231 and SUM-159 models (35). The current study supports echinomycin’s reemergence as an immunotherapeutic agent.

Targeting HIF-1α in immunotherapy is a relatively new concept, for which proof of principle has been amply demonstrated by others (12, 23, 27). While our studies expand on these early studies that established the significance of the HIF-1α pathway in immunosuppression, they allow us to propose what we believe is a new paradigm: exploiting differential regulatory pathways of PD-L1 expression simultaneously can uncouple immunotherapeutic effects and irAEs. In this study, we demonstrate that targeting HIF-1α can achieve such an effect in preclinical models of cancer. These findings provide what we believe is a new perspective for immunotherapy drug development.

Whether hypoxia/HIF-1α pathways are pro- or antiinflammatory is a topic of debate. Sitkovsky’s group originally demonstrated the first in vitro evidence that hypoxia is immunosuppressive for T cells (36), and the first in vivo genetic evidence that hypoxia is immunosuppressive for T and B cells of the adaptive immune system (37). Subsequently, it was shown that direct elimination of TME hypoxia can improve cancer immunotherapy in mice (27, 38, 39). On the other hand, studies by Johnson and colleagues found HIF-1α to be essential for myeloid cell–mediated inflammation (40). In T cells, HIF-1α is transiently stabilized in T cells following TCR activation (41), and plays an important role in regulating Treg and Th17 balance (26). According to Doedens et al., HIFs enhance the effector responses of CD8^+^ T cells to persistent antigen (42). Palazon et al. showed that selective HIF-1α targeting in T cells inhibited IFN-γ and granzyme B production in CD8^+^ TILs (43). Notably, echinomycin treatment had the opposite effect. Therefore, abrogating HIF-1α–mediated immune suppression in tumor cells and tumor-associated myeloid cells may be more important for immunotherapeutic efficacy than preserving HIF-1α function in T cells.

The pioneering work in developing immunotherapy targeting PD-1 and PD-L1 (13, 44–47) has led to the most important breakthrough in cancer therapy, with rapidly expanding indications of anti–PD-1/PD-L1 antibodies adopted for treatment of both hematological and nonhematological malignancies (48). However, the current approach that overcomes tumor evasion of host immunity also disables the immune tolerance checkpoint, leading to significant irAEs, particularly when used in conjunction with anti–CTLA-4 antibodies. Here, we showed that targeting HIF-1α not only overcomes immune evasion in the TME, but also fortifies the immune tolerance checkpoint in normal tissues.

HIF-1α is generally inactivated in normal tissues but frequently stabilized in tumor cells regardless of oxygen tension (49, 50). This fundamental difference allows us to selectively inhibit PD-L1 expression in the TME using LEM. Surprisingly, LEM induced PD-L1 expression in normal tissues of immunocompetent mice, including liver, kidney, and colon. The unexpected induction of PD-L1 was attributable to elevated IFN-γ production associated with LEM-induced expansion of IFN-γ–producing T cells, including Tc1 and Th1 cells. The induced PD-L1 is causatively associated with reduction in inflammation and intestinal leakage induced by anti–CTLA-4 antibodies, as it is abrogated by anti–PD-1 antibodies.

The ability of anti–PD-1 to abrogate protection by echinomycin also suggests an interesting explanation for how anti–PD-1 exacerbates irAEs when used in conjunction with anti–CTLA-4; PD-L1 is induced by anti–CTLA-4–induced IFN-γ as a negative feedback mechanism to control irAEs. By preventing PD-L1 from interacting with PD-1, anti–PD-1/PD-L1 antibodies exacerbate irAEs caused by anti–CTLA-4 antibodies. In contrast to anti–PD-1, LEM not only further enhanced anti–CTLA-4–induced PD-L1 in normal tissue, but also allowed PD-L1 to signal through PD-1 to supercharge the immune tolerance checkpoint function.

While HIF-1α has been shown to be involved in degradation of Foxp3 and induce the Th17 phenotype (26), its function in inducing the Th1 phenotype has also been reported (51). Our data presented herein show a strong effect of LEM in inducing IFN-γ–producing cells, including Tc1 and Th1 cells. It is unclear whether LEM promotes Tc1 expansion in vivo by cell-intrinsic targeting of HIF-1α or indirectly by a reduction in regulatory T cells. Regardless of whether the effect is T cell intrinsic, the induction of PD-L1 in normal tissues is tissue-cell extrinsic. In contrast, in cancer cells, targeting HIF-1α resulted in a cell-intrinsic inhibition of PD-L1. Thus, the data presented herein revealed a cancer cell–intrinsic inhibition of PD-L1 and normal tissue cell–extrinsic induction of PD-L1 by LEM. Together, these 2 activities provide what we believe is the first example in cancer immunotherapy of an approach that abrogates the PD-1–PD-L1 checkpoint in the TME to eliminate immune evasion by cancer cells, while fortifying its immune tolerance checkpoint activity in normal tissues. Therefore, HIF-1α inhibitors represent an ideal partner for CTLA-4–targeted immunotherapy.

## Methods

### Cell lines.

4T1, E0771, and MC38 cells were obtained from American Type Culture Collection.

### Therapeutic agents.

Echinomycin was formulated with liposomes as previously described (35). Recombinant ipilimumab was provided by Lakepharma Inc. Remaining therapeutic antibodies were from BioXCell as follows: anti–mouse CTLA-4, clone 9D9 (BE0164); anti–mouse PD-1, clone RMP1-14 (BE0146); anti–mouse IFN-γ, clone XMG1.2 (BE0055); anti–mouse CD4, clone GK1.5 (BE0003-1); anti–mouse CD8α, clone YTS 169.4 (BE0117); and anti–mouse NK1.1, clone PK136 (BE0036).

### Flow cytometry.

Data were acquired on BD FACSCanto II or Cytek Aurora and analyzed using FlowJo software (v10.7.0). A detailed description of the fluorescent antibodies used are provided in supplemental methods.

### Mice.

BALB/cAnNCr and C57BL/6NCr were obtained from the National Cancer Institute, and NOD.Cg-*Prkdc^scid^Il2rγ^tm1Wjl^*/SzJ (NSG) mice were purchased from the University of Maryland School of Medicine and bred in-house. B6.129-*Hif1a^tm3Rsjo^*/J and Tg(Cd4-cre)1Cwi/BfluJ mice were from The Jackson Laboratory. *CTLA4*^h/h^-KI mice were generated and bred in-house and have been previously described (30).

### Tumor models.

The details of each experiment are specified in the figure legends. Tumor cells were suspended in RPMI-1640 medium and injected into recipient mice at 0.5 × 10^6^ to 1.0 × 10^6^ cells/50 μL/mouse. 4T1 and E0771 cells were injected orthotopically into the first (left) mammary fat pad of female recipients; MC38 cells were injected into the flank. On day 6 after transplantation, mice were assigned to treatment groups such that comparable initial mean tumor volumes between experimental and control groups were achieved. Tumor volumes were calculated using the formula *V* = *ab*^2^/2, where *a* is the longer diameter and *b* is the shorter diameter. LEM, or equivalent empty liposomes as vehicle control, were administered by intravenous (i.v.) injection into the lateral tail vein on the indicated days, at 0.15–0.25 mg/kg. Intraperitoneal (i.p.) injection was used to deliver therapeutic antibody 9D9, RMP1-14, or XMG1.2 at 0.2 mg/mouse/injection. The mice from different groups were sacrificed at the same time points for analyses.

### GI irAE model.

Ten-day-old *CTLA4*^h/h^ mice received 0.1 mg of ipilimumab i.p. on days 10, 13, 16, and 19 after birth and the FITC-dextran assay was performed on day 32 to detect GI irAEs. On day 33, the mice were euthanized for flow cytometry and histological analyses described in the figures. LEM (10 μg/kg), ipilimumab (0.1 mg/mouse/injection), RMP1-14 (0.2 mg/mouse/injection), and XMG1.2 (0.2 mg/mouse/injection) were administered i.p. according to the schedule in Figure 8A. See supplemental methods for additional details.

### Statistics.

All experiments were replicated at least twice with similar results. Appropriate statistical tests were selected on the basis of whether the data with outlier deletion was normally distributed by using the D’Agostino & Pearson normality test. Data comparing 2 groups were analyzed by unpaired, 2-tailed Student’s *t* test. Unless otherwise noted in the figure legends, 1-way analysis of variance (ANOVA) with Sidak’s post hoc test was used for multiple comparisons, and 2-way ANOVA for analysis of tumor kinetics. The correlation coefficient and *P* value for linear regression were calculated by Pearson’s method. Sample sizes were chosen with adequate statistical power on the basis of the literature and past experience. In the graphs, data are shown as mean ± SEM, indicated by horizontal line and *y*-axis error bars, respectively. Statistical calculations were performed using Prism 8 software (GraphPad Software). NS in the figures indicates no significant difference. A *P* value of less than 0.05 was considered significant: *P < 0.05, **P < 0.01, ***P < 0.001, ****P < 0.0001.

### Study approval.

All animal experiments were conducted according to guidelines established by the NIH *Guide for the Care and Use of Laboratory Animals* (National Academies Press, 2011). All procedures involving experimental animals were approved by the Institutional Animal Care and Use Committee (IACUC) of the University of Maryland School of Medicine.

## Author contributions

CMB, Yan Liu, and YW designed and performed research as well as prepared the manuscript. ML and XD performed research and helped with methodology. MD and PZ provided advice on experimental design and helped with histological experiments and editing. Yang Liu and YW designed the study, analyzed data, supervised the study, and wrote the manuscript.

## Supplementary Material

Supplemental data

## Version 1 03/03/2022

In-Press Preview

## Version 2 05/02/2022

Electronic publication
