# Supplementary material for: Targeting HIF-1α abrogates PD-L1–mediated immune evasion in tumor microenvironment but promotes tolerance in normal tissues
Source: J Clin Invest. 2022 May 2;132(9):e150846. doi: 10.1172/JCI150846 (PMC9057613; doi:10.1172/JCI150846)

Supplemental Figures

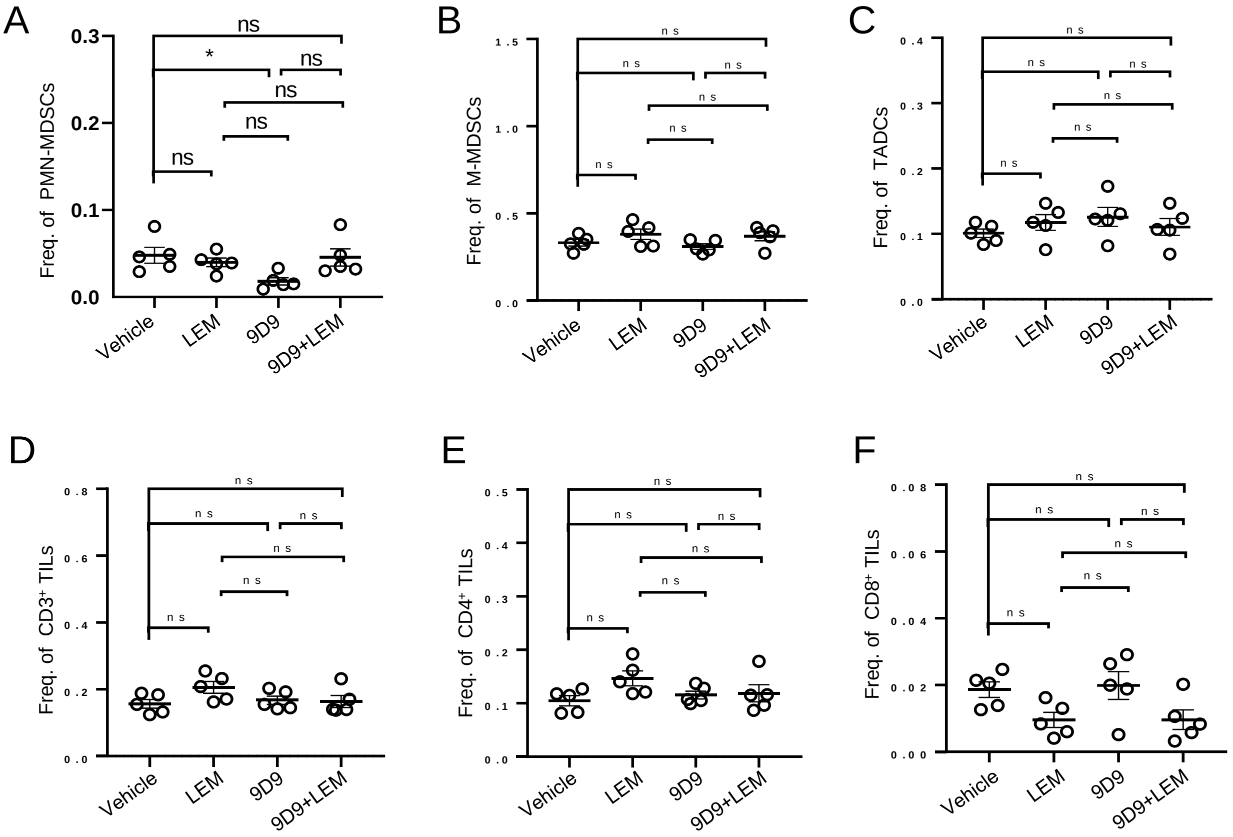

Figure S1. Frequencies of immune subsets within the E0771 tumors. Extension to Figure 5 in main text. The frequencies of tumor-infiltrated myeloid cells and tumor-infiltrated lymphocytes (TILs) are shown, expressed as the ratio among total hematopoietic cells (gated on Live_CD45$^+$) for mice treated with vehicle, echinomycin (LEM), anti-CTLA-4 (9D9), or combination. **A.** Frequency of PMN-MDSCs. **B.** Frequency of M-MDSCs. **C.** Frequency of CD11b$^+$CD11c$^+$ TAMs. **D.** Frequency of CD3$^+$ TILs. **E.** Frequency of CD4$^+$ TILs. **F.** Frequency of CD8$^+$ TILs. Statistics were performed by one-way ANOVA with Sidak’s multiple comparisons test.

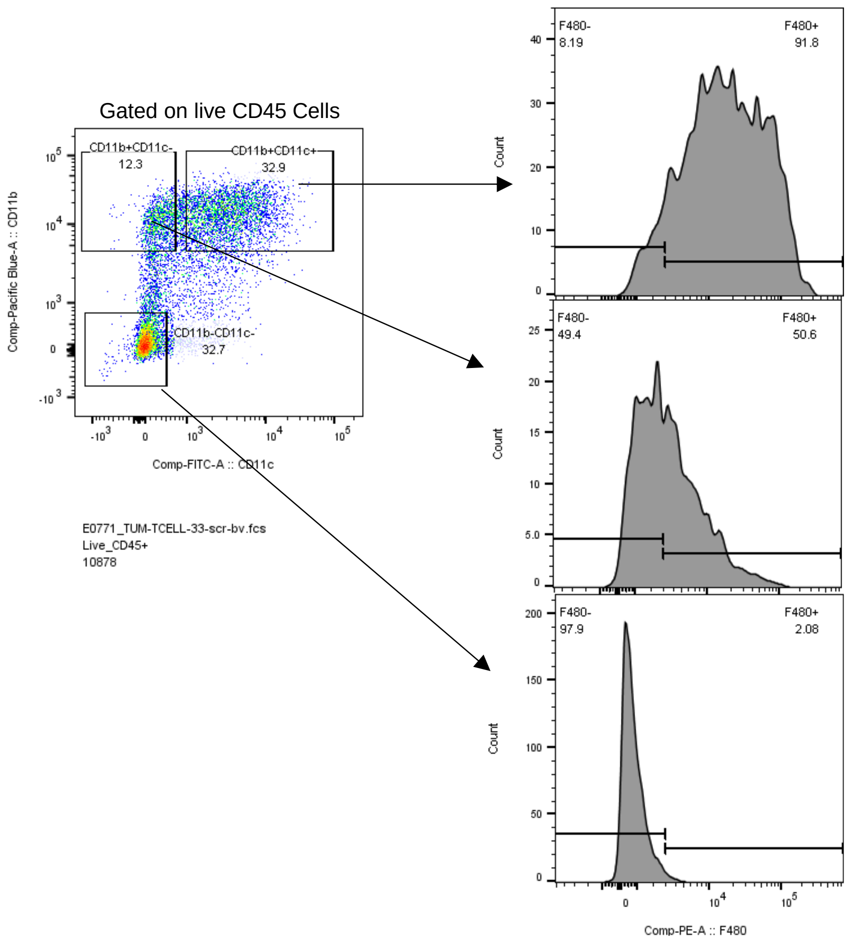

Figure S2. The majority of CD11b⁺CD11c⁺ tumor-infiltrated myeloid cells within E0771 tumors co-express F4/80 macrophage marker. Extension to Figure 5 in main text. The gating strategy for the identification of tumor-infiltrated myeloid cells (CD11b$^+$), divided into CD11c$^+$ or CD11c$^-$ populations is shown. The majority (>90%) of CD11b$^+$CD11c$^+$ cells co-express F4/80, indicated by the histogram. F4/80 expression in CD11b$^+$CD11c$^-$ and CD11b$^-$CD11c$^-$ cells are also shown.

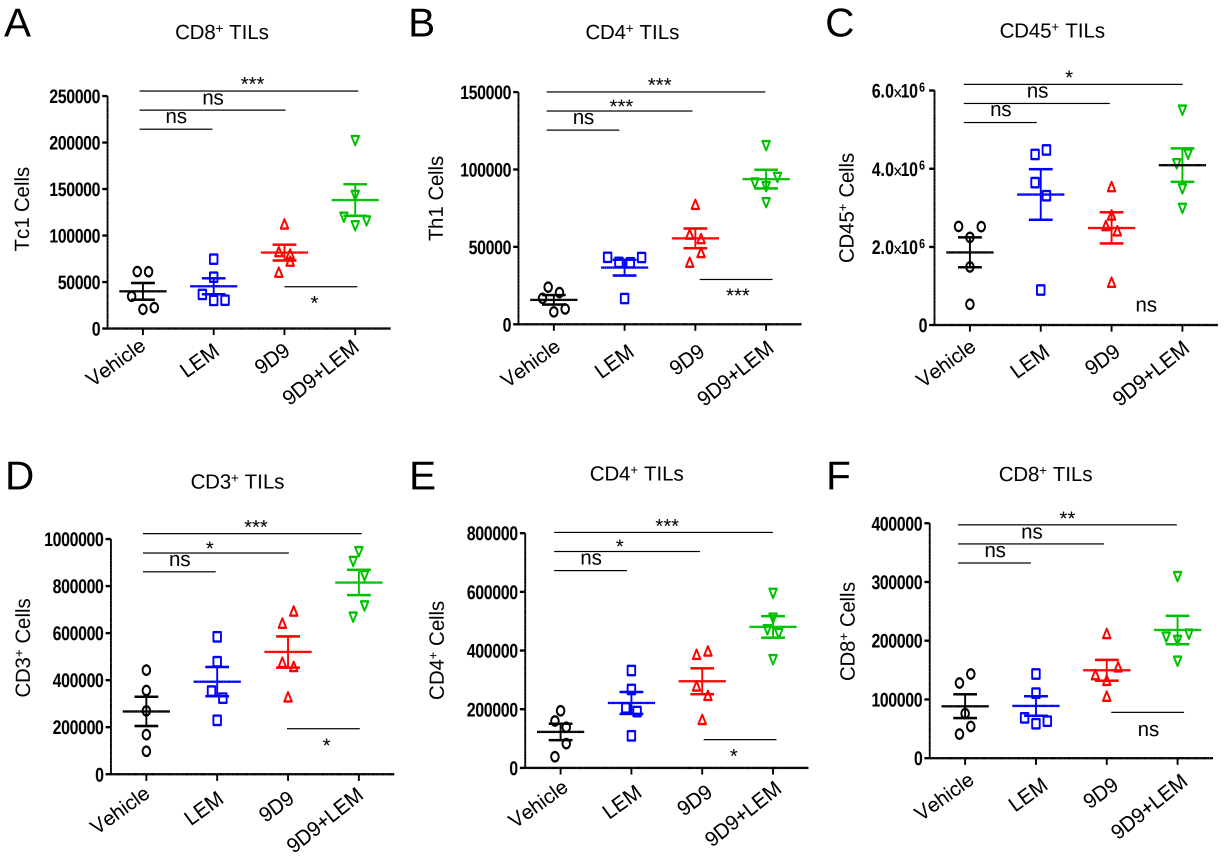

Figure S3. Absolute numbers of TIL subsets in E0771 tumors. E0771 cells (0.7x106) were orthotopically transplanted into C57BL/6 mice (day 0). The mice received treatment with vehicle, echinomycin (0.25 mg/kg/dose), 9D9 (0.2 mg/mouse/injection), or their combination on days 6, 8, and 10, and were euthanized on day 14. The tumors were weighed, then dissociated for analysis of TIL subsets by flow cytometry. Cell counts were acquired by flow cytometry and normalized to tumor weights, shown in the y axes as absolute cell count per gram of tumor. Data are shown for one experiment (n=5/group), representative of two experiments.

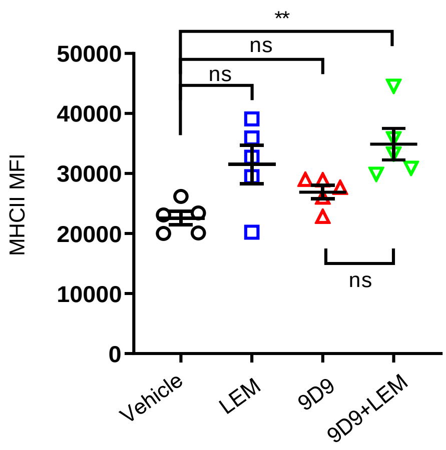

Figure S4. Expression of MHCII on CD11c+ TAMs in E0771-bearing mice treated with LEM and/or 9D9. E0771 cells (0.5x106) were orthotopically transplanted into C57BL/6 mice (day 0). The mice received treatment with vehicle, echinomycin (0.25 mg/kg/dose), 9D9 (0.2 mg/mouse/injection), or their combination on days 6, 8, 10, and 12, and were euthanized on day 14. The tumors were dissociated analyzed by flow cytometry to quantitate MHCII expression on CD11c+ TAMs (gated on Singlets/Live_CD45+/CD11b+CD11c+). The upper panels show representative histograms depicting I-Ab expression among TAM, while the lower panel shows mean ± SEM of MFI. Statistics analyzed by one-way ANOVA with Sidak’s posttest.

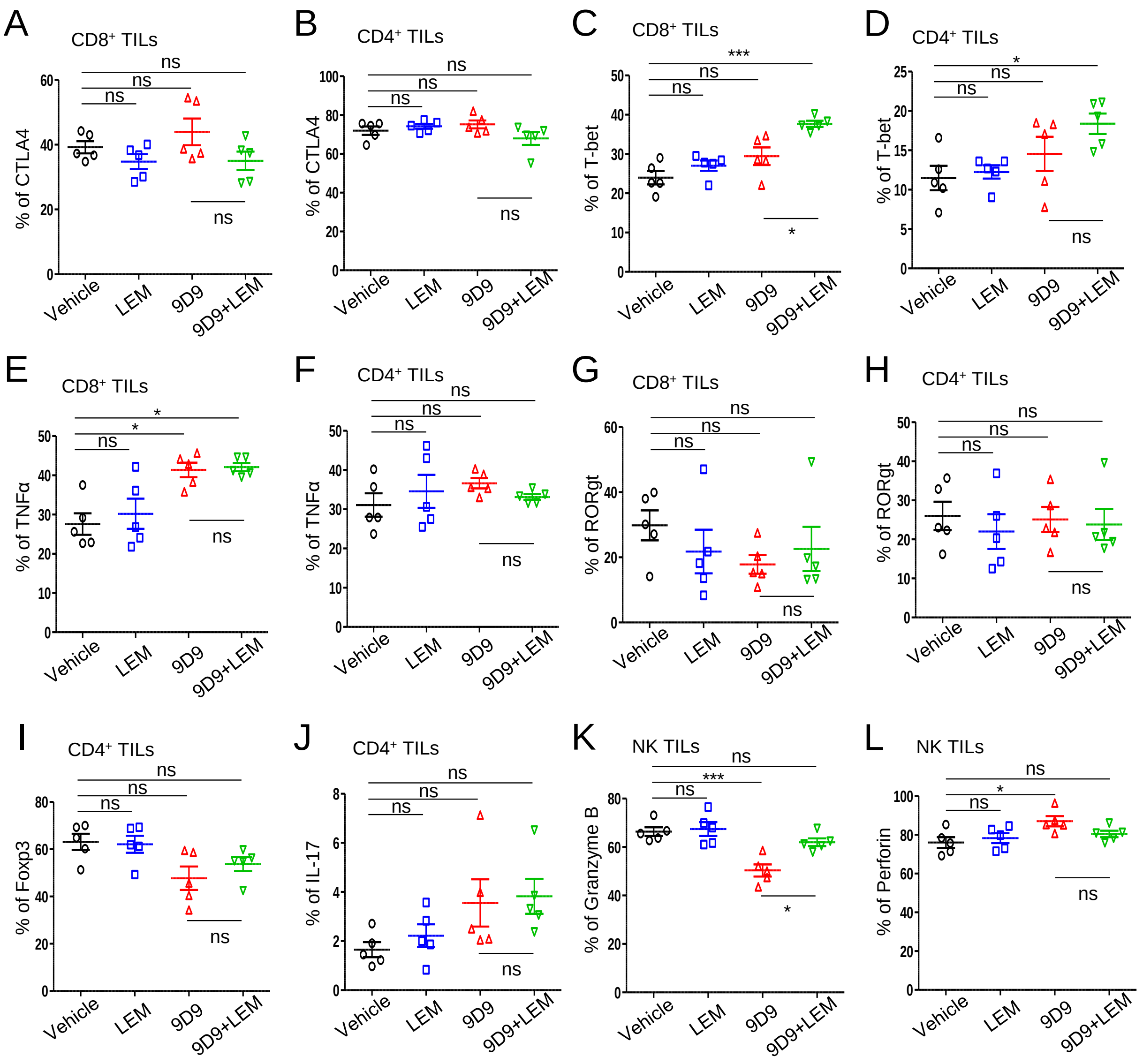

Figure S5. TIL expression of lineage-specific cytokines, transcription factors, and cytolytic effector molecules in E0771 mice treated with LEM and/or 9D9. E0771 cells (0.7x10^6) were orthotopically transplanted into C57BL/6 mice (day 0). The mice received treatment with vehicle, echinomycin (0.25 mg/kg/dose), 9D9 (0.2 mg/mouse/injection), or their combination on days 6, 8, and 10, and were euthanized on day 14 for flow cytometric analysis of CD4 (gated on Singlets/Live_$CD45^+$/$CD3^+$/$CD8^-CD4^+$) and CD8 (gated on Singlets/Live_$CD45^+$/$CD3^+$/ $CD8^+CD4^-$) TILs. The frequencies of indicated subsets are plotted on the y axes expressed as

mean ± SEM of the respective CD4 or CD8 parent subsets. **A-B.** CTLA-4 expression in CD8+ and CD4+ TILs, respectively. **C-D.** T-bet expression in CD8 and CD4 TILs. **E-F.** TNF-a expression in CD8 and CD4 TILs. **G-H**. RORγt expression in CD8 and CD4 TILs. **I**. Frequency of Treg cells (gated on Singlets/Live_CD45^{+}/CD3^{+}/CD8^{-}CD4^{+}/Foxp3^{+}) among total CD4. **J.** Frequency of Th17 cells (gated on Singlets/Live_CD45^{+}/CD3^{+}/CD8^{-}CD4^{+}/IL17A^{+}) among total CD4. **K-L**. Granzyme B and perforin expression in tumor infiltrating NK cells (gated on Singlets/Live_CD45^{+}/NK1.1^{+}). Data are shown for one experiment (n=5/group), representative of two experiments. Statistics analyzed by one-way ANOVA with Sidak's posttest.

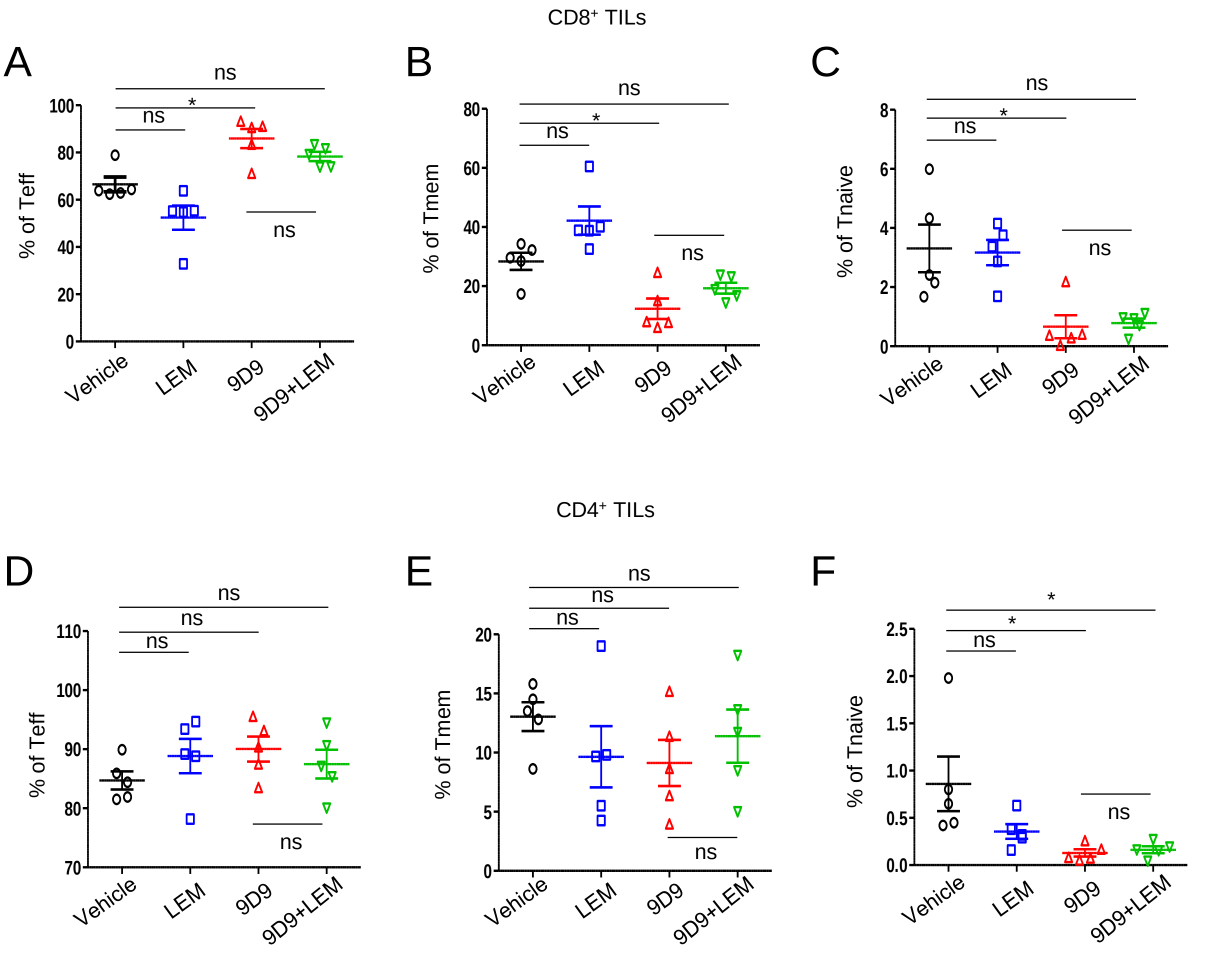

Figure S6. Expression of activation markers on TILs from E0771-bearing mice treated with LEM and/or 9D9. E0771 cells (0.7x106) were orthotopically transplanted into C57BL/6 mice (day 0). The mice received treatment with vehicle, echinomycin (0.25 mg/kg/dose), 9D9 (0.2 mg/mouse/injection), or their combination on days 6, 8, and 10, and were euthanized on day 14 for flow cytometric analysis of CD4 (gated on Singlets/Live_$CD45^+$/$CD3^+$/$CD8^-CD4^+$) and CD8 (gated on Singlets/Live_$CD45^+$/$CD3^+$/ $CD8^+CD4^-$) TILs. **A-C**. Proportions of CD8 Teff ($CD44^+CD62L^-$), Tmem ($CD44^+CD62L^+$), and Tnaive ($CD44^-CD62L^-$) among total CD8, expressed as the mean ± SEM. **D-F.** As in A, except the analysis is for CD4 TILs. Data are shown for one experiment (n=5/group), representative of two experiments. Statistics analyzed by one-way ANOVA with Sidak's posttest.

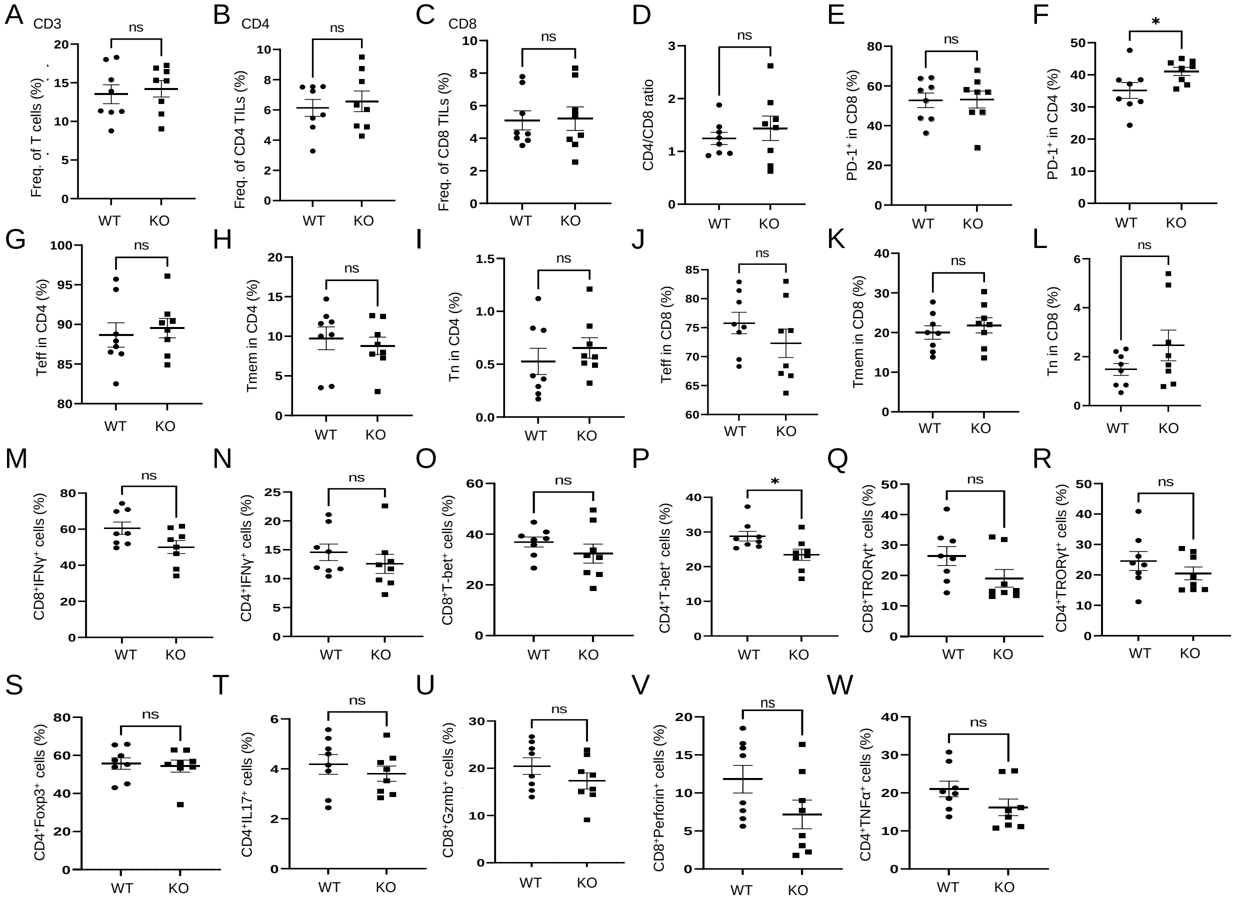

Figure S7. Comparison of TILs properties in E0771 model in mice with T cell-intrinsic Hif1α knockout vs wild type littermates E0771 cells (0.7×10^6) were orthotopically transplanted into CD4-cre(+) Hif1a$^{fl/fl}$ (KO) mice or CD4-cre(-) (WT) littermates. On day 14 after tumor cell injection, the tumors were processed for flow cytometry. For secreted markers, the tumor cell suspensions containing lymphocytes were stimulated with PMA+Ionomycin with GolgiStop for 4hrs. A. Frequency of T cells (gated on Singlets/Live_CD45$^+$/CD3$^+$) among total Live CD45$^+$ cells. B. Frequency of CD4 TILs (gated on Singlets/Live_CD45$^+$/CD3$^+$/CD4$^+$CD8$^-$) among total Live CD45$^+$. C. Frequency of CD8 TILs (gated on Singlets/Live_CD45$^+$/CD3$^+$/CD4$^-$CD8$^+$). **D**. Ratio of CD4 to CD8 TILs. **E**. Frequency of PD-1$^+$ CD8 TILs among total CD8 TILs. **F**. Frequency of PD-1$^+$ CD4 TILs among total CD4 TILs. G-I. The frequencies of CD8 Teff

($CD44^{+}CD62L^{-}$), Tmem ($CD44^{+}CD62L^{+}$), and Tnaive ($CD44^{-}CD62L^{-}$) among total CD8. **J-L.** As in G-I, except the analysis is for CD4 TILs. The frequencies of cells among total CD8 TILs (gated on Singlets/Live_$CD45^{+}$/$CD3^{+}$/$CD4^{-}CD8^{+}$). **M**. Frequency of IFNγ+ CD8 TILs among total CD8 TILs. **N**. Frequency of $IFNg^{+}$ CD4 TILs among total CD4 TILs. **O**. Frequency of T-bet^{+} CD8 TILs among total CD8 TILs. **P**. Frequency of T-bet^{+} CD4 TILs among total CD4 TILs. **Q**. Frequency of $ROR\gamma t^{+}$ CD8 TILs among total CD8 TILs. **R**. Frequency of $ROR\gamma t^{+}$ CD4 TILs among total CD4 TILs. **S**. Frequency of $Foxp3^{+}$ CD4 TILs among total CD4 TILs. **T**. Frequency of $IL17^{+}$ CD4 TILs among total CD4 TILs. **U**. Frequency of granzyme B^{+} CD8 TILs among total CD8 TILs. **V**. Frequency of $perforin^{+}$ CD8 TILs among total CD8 TILs. **W**. Frequency of $TNF\alpha^{+}$ CD4 TILs among total CD4 TILs. All data are expressed as the mean±SEM. Data are shown for one experiment (n=5/group), representative of two experiments. Statistics analyzed by one-way ANOVA with Sidak's posttest.

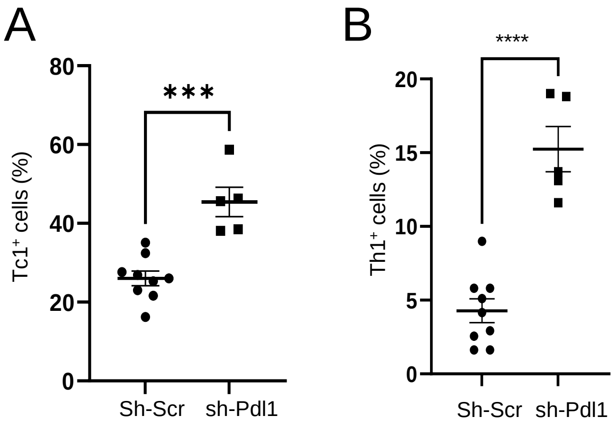

Figure S8. E0771 with scrambled shRNA (sh-Scr) or shRNA for Pdl1 (sh-Pdl1) were orthotopically transplanted $0.5x10^6$ cells per mouse into C57BL/6 mice (day 0). On day 14 after tumor cell injection, the mice were euthanized for flow cytometry analysis of the TILs stimulated for 4 hrs in vitro. Shown are the frequencies of Tc1 (A) or Th1 (B) cells among total CD8 or CD4 TILs, respectively. The data are expressed as the mean ± SEM and are representative of two experiments. Statistics analyzed by unpaired student's ttest (unpaired).

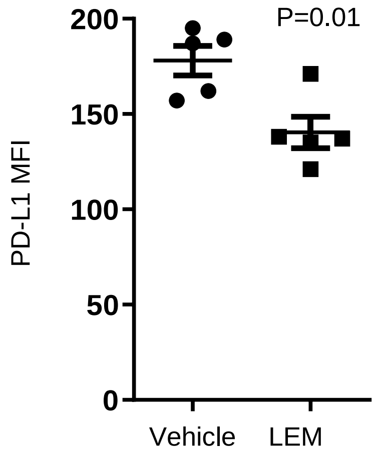

Figure S9. Effect of LEM on PD-L1 expression in tumors of *Hif1a*$^{fl/fl}$CD4-cre(+) E0771 mice. *Hif1a*$^{fl/fl}$CD4-cre(+) mice received E0771 cells ($0.5x10^{6}$) on day 0, followed by treatment with vehicle or LEM (0.25 mg/kg, once on days 6, 8, and 10). On day 14, the mice were euthanized for flow cytometry analysis of PD-L1 expression on tumor cells (single cells/Live/$CD45^{-}EGFP^{+}$). Data shown as mean fluorescence intensity (MFI) ± SEM for PD-L1 staining and are pooled from two experiments (n=10 mice/group), and statistics were determined by two-tailed student's ttest (unpaired).

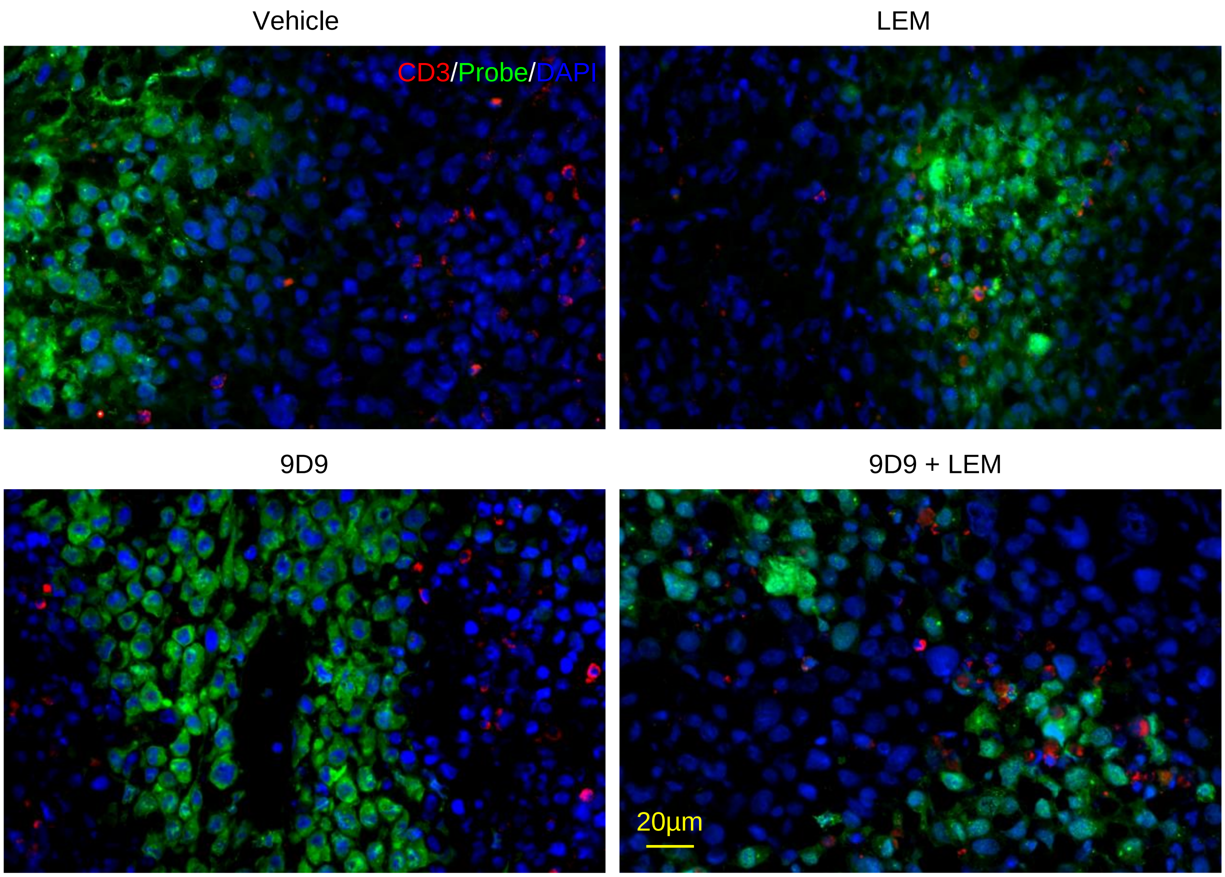

Figure S10. E0771 bearing mice received hypoxyprobe (pimonidazole) 90 minutes prior to euthanasia according to the manufacturer's instructions. The tumor tissues were then frozen and sectioned and stained with the hypoxyprobe-1 kit for anti-pimonidazole (Probe, green) or anti-mouse CD3 (red), counterstained with DAPI.

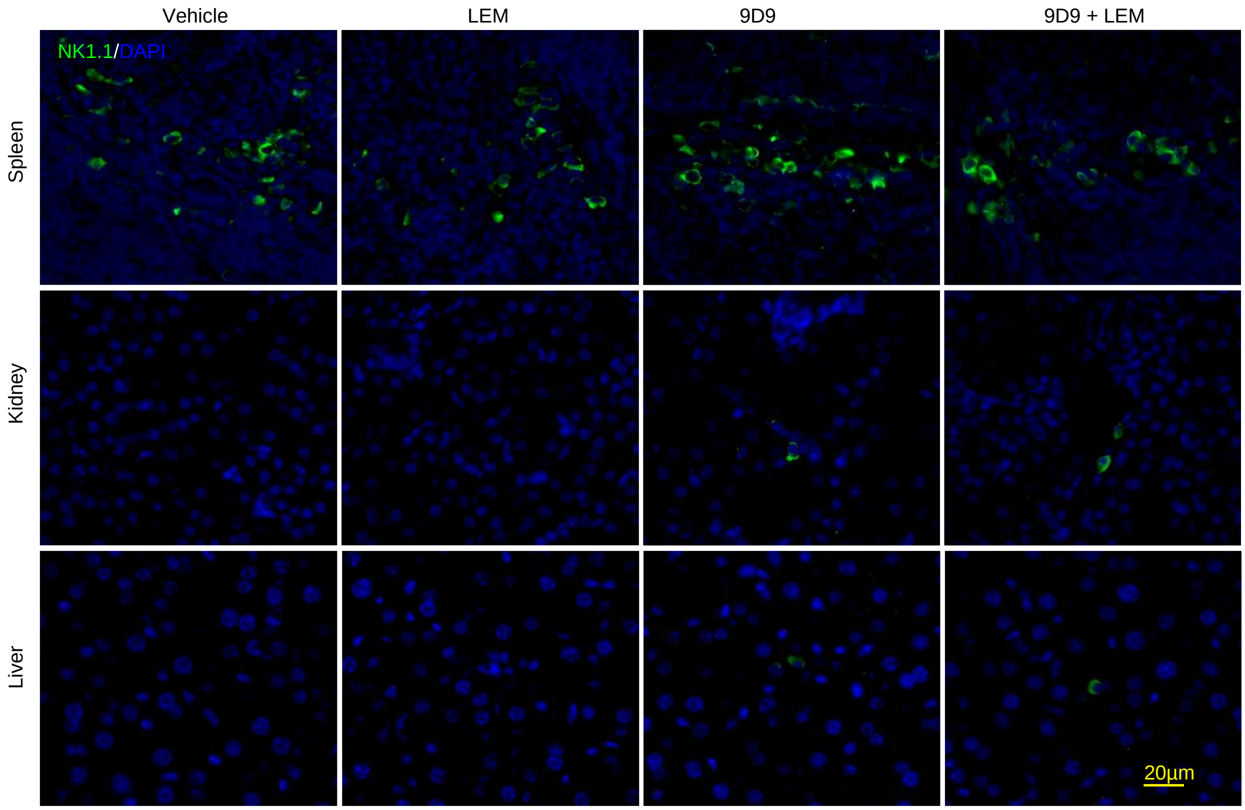

Figure S11. NK cell infiltration into normal tissues in response to 9D9 or LEM Extension to Figure 7 in the main text. Representative immunofluorescence staining is shown for NK1.1 in the kidney and liver tissues of E0771-bearing mice treated with vehicle, LEM, 9D9, or 9D9 + LEM. Spleen tissue shown as positive control. The panels with positive stains for kidney and liver highlight rare incidences of NK cells infiltrated. Most fields of view were negative. The data are representative of each group, with n=5 mice per group.

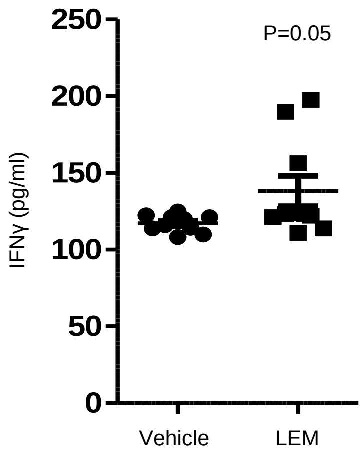

Figure S12. Serum IFNγ concentrations and flow cytometry staining of liver PD-L1 in E0771 mice. C57BL/6 mice bearing E0771 tumors and treated with vehicle or LEM (n=10/group) were bled on day 13 after tumor cell injection to extract serum, followed by euthanasia on day 14 for harvesting of liver tissues. The serum IFNγ concentrations are shown, as analyzed by cytometric bead array and flow cytometry. Data are pooled from two independent experiments, shown as the mean ± SEM, and analyzed by two-tailed student's ttest (unpaired).

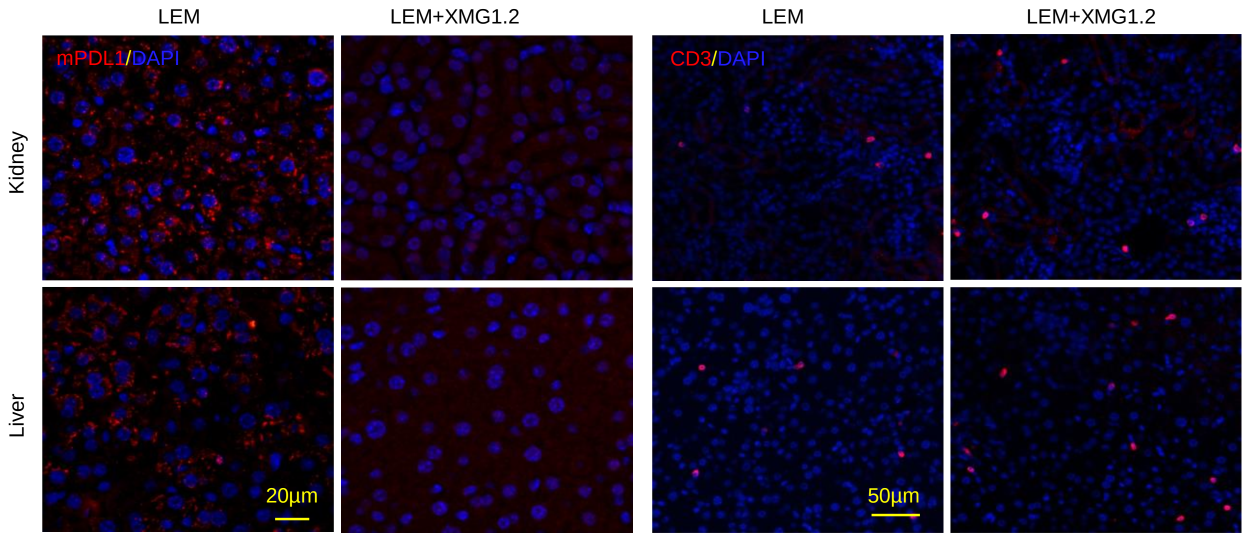

Figure S13. Extension to Figure 7 in the main text: PDL1 expression in kidney and liver induced by LEM also depend on IFNγ. Representative immunofluorescence staining is shown for PDL1 or CD3 in the kidney and liver tissues of E0771-bearing mice treated with LEM alone, or in combination with XMG1.2, based on the same experimental conditions described in Figure 7.

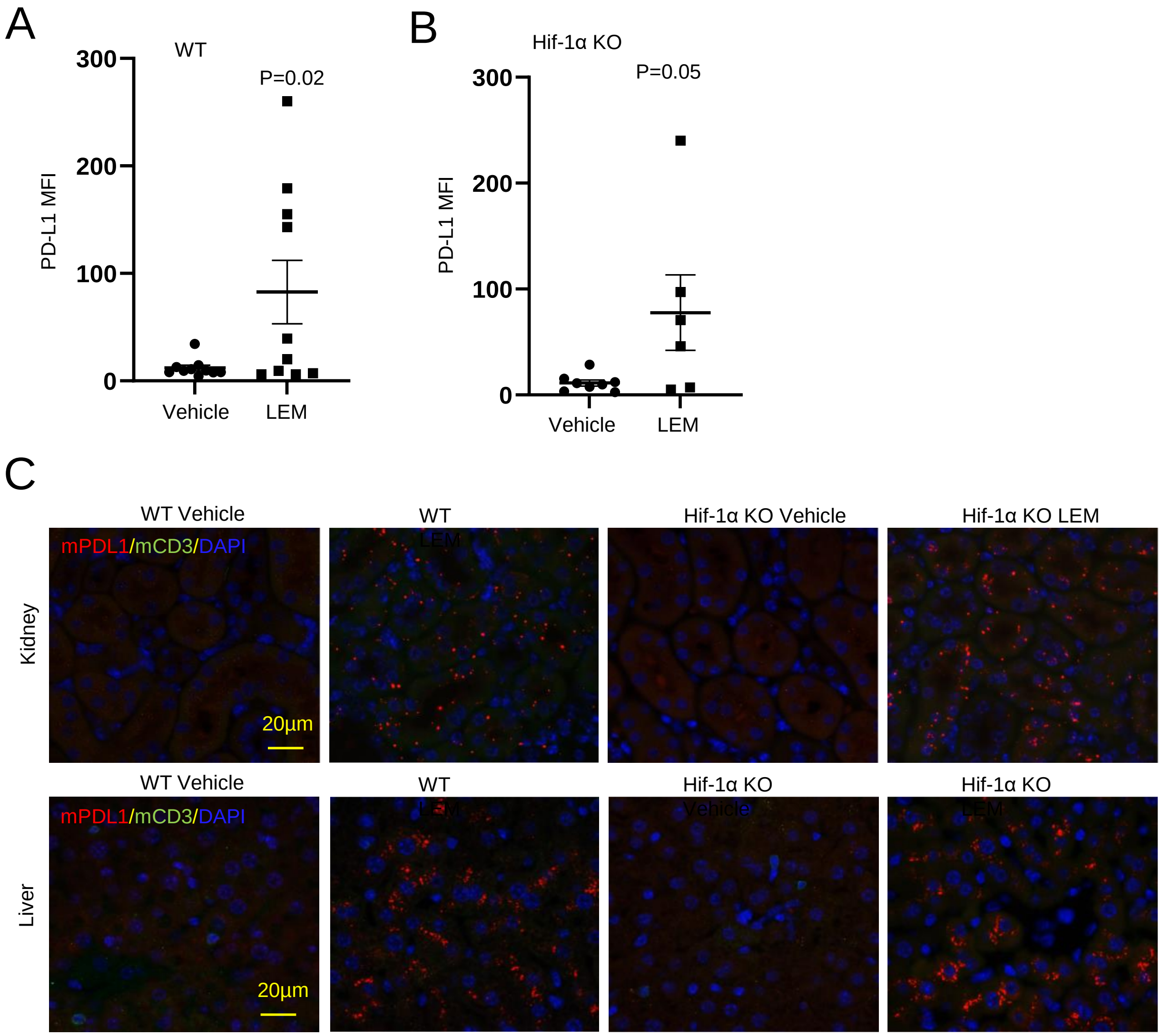

Figure S14. Effect of LEM on PD-L1 expression in *Hif1a*$^{fl/fl}$CD4-cre(+) E0771 mice. *Hif1a*$^{fl/fl}$CD4-cre(-) (WT) or (+) (KO) mice received E0771 cells (0.5x10^6) on day 0, followed by treatment with vehicle or LEM (0.25 mg/kg, once on days 6, 8, and 10), and on day 14, the mice were euthanized. A and B. Flow cytometry analysis of PD-L1 expression in liver tissues (single cells/Live/$CD45^-$), for Hif1α WT (A) or KO (B) mice. Data shown as mean fluorescence intensity (MFI) ± SEM for PD-L1 staining and are pooled from two experiments (n=10 mice/group), and statistics were determined by two-tailed student's ttest (unpaired). **C.**

Representative immunofluorescence staining of PDL1 and CD3 in the kidney and liver tissues of WT or KO mice, treated with vehicle or echinomycin.

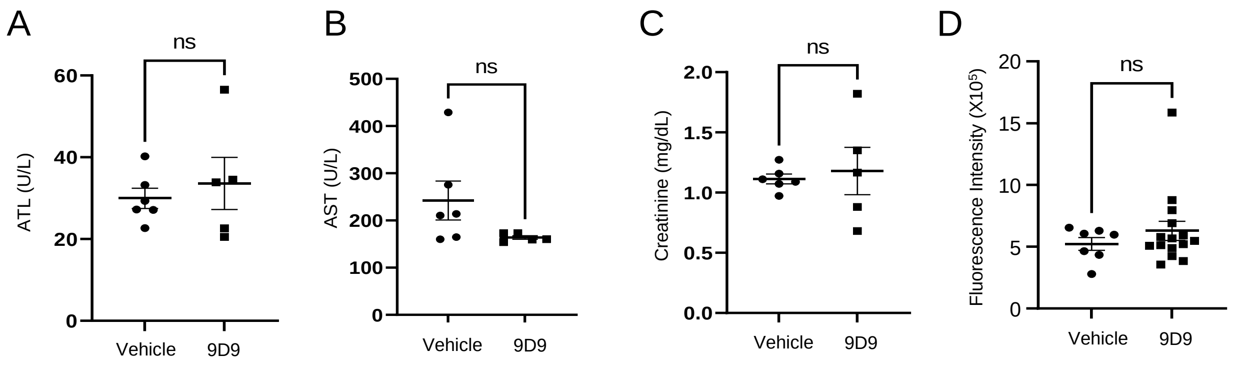

Figure S15. Measurements of serum biomarkers for irAE in the adult tumor-bearing mouse. Serum levels of liver enzymes (ALT and AST), creatinine, and FITC-dextran were analyzed for readout of hepatotoxicity, nephrotoxicity, or gastrointestinal irAE, respectively, in response to 9D9 treatment. **A-C** Adult C57BL/6 mice were inoculated with E0771 cells and began receiving therapies on day 6, when tumors averaged 3x3-5x5. Either isotype control (n=6, vehicle) or 9D9 (n=5) was given ip to the mice at 200 ug/mouse, on days 6, 8, 10, 12, 16, 18, and 20, and the serum was collected from the mice on day 24 to measure liver enzymes or creatinine. **D** The same protocol was followed for FITC dextran assay, with measurement on day 26 (vehicle, n=6; 9D9, n=15). Statistics were determined by two-tailed student's ttest (unpaired).

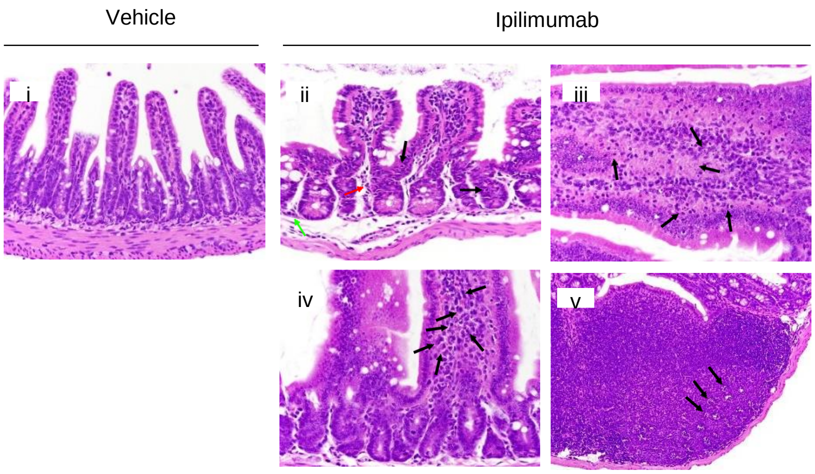

Figure S16. Ipilimumab induced intestinal inflammation. Representative H&E images from the intestines of mice treated with either vehicle (panel i) or Ipilimumab (panels ii-v). Panel i: Ileum, 40X, normal pathology; panel ii: jejunum, 40X, mild-moderate infiltration of lymphocytes, neutrophils (red arrows), plasma cells (green arrows), and mucosal mast cells (black arrows); panel iv: duodenum, 40X, cell debris in lamina propria; panel v: Peyer's patch, 20X, active germinal centers with apoptotic foci (arrows).

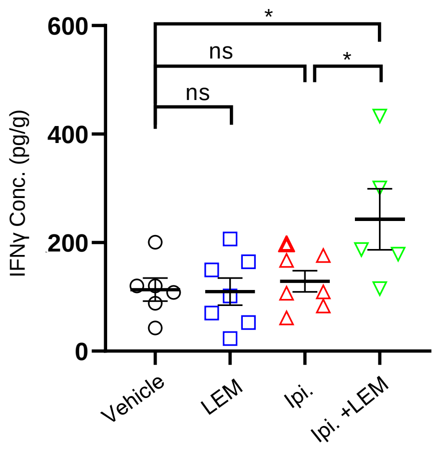

Figure S17. IFNγ levels in intestinal tissues of *CTLA4*$^{h/h}$ mice treated with echinomycin and/or Ipilimumab. CTLA4h/h mice were treated with vehicle, echinomycin, Ipilimumab, or Ipilimumab + echinomycin as in Figure 7E. On day 32, IFNγ levels were measured in the small intestine by cytometric bead array. The data is expressed as amount of IFNγ measured, normalized to the weight of the tissue collected. Statistics analyzed by one-way ANOVA with Sidak's posttest. Pooled data is shown from two experiments (n=2-3 mice/group/experiment).

Supplemental methods.

Antibodies (Flow Cytometry) Antibodies used for flow cytometry were as follows (cat. Number): BD Biosciences (San Jose, CA): BUV496 anti-mouse CD45, clone 30-F11 (749889); BUV480 anti-mouse CD11b, clone M1/70 (566117); BUV805 anti-mouse CD4, clone GK1.5 (612900); BUV563 anti-mouse CD8a, clone 53-6.7 (748535); BUV395 anti-mouse CD279, clone J43 (744549); FITC anti-mouse CD11c, clone HL3 (553801). BioLegend (San Diego, CA): PerCP/Cy5.5 anti-mouse NK1.1, clone PK136 (108728); SparkBlue-550 anti-mouse CD3, clone 17A2 (100260); PE/Cy5 anti-mouse CD44, clone IM7 (103010); AlexaFluor-700 anti-mouse CD62L, clone MEL-14 (104426); BrilliantViolet-605 anti-mouse CD152, clone UC10-4B9 (106323); PE anti-mouse perforin, clone S16009B (154406); FITC anti-mouse granzyme B, clone QA16A02 (372206); AlexaFluor-647 anti-mouse IL-17A, clone TC11-18H10.1 (506912); BrilliantViolet-421 anti-mouse IL-2, clone JES6-5H4 (503825); BrilliantViolet-711 anti-mouse TNF-α, clone MP6-XT22 (506349); BrilliantViolet-785 anti-mouse T-bet, clone 4B10 (644835); APC-Cy7 anti-mouse I-A/I-E, clone M5/114.15.2 (107628); PerCP anti-mouse CD3ε, clone 145-2C11 (100326); PerCP/Cy5.5 anti-mouse CD45, clone 30-F11 (103132); PE/Cy7 anti-mouse Ly-6G, clone 1A8 (127618); APC anti-mouse Ly-6C, clone HK1.4 (128016); FITC anti-mouse NKp46, clone 29A1.4 (137606). eBioscience (San Diego, CA): eFluor-450 anti-mouse Foxp3, clone FJK-16s (48-5773-82); APC anti-mouse IFNγ, clone XMG1.2 (17-7311-82); PE-Cy7 anti-mouse RORγt, clone B2D (25-6981-82); PE Rat IgG2a kappa Isotype Control, clone eBr2a (12-4321-83); PE anti-mouse CD274, clone MIH5 (12-5982-82); PE anti-mouse CD3ε, clone 145-2C11 (12-0031-85; eFluor-450 anti-mouse CD3, clone 17A2 (48-0032-82); APC anti-mouse Foxp3, clone FJK-16s (17-5773-82); APC anti-mouse CD45, clone 30-F11 (17-0451-83); PE-Cy7 anti-mouse CD4, clone GK1.5 (25-0041-82); FITC anti-mouse CD8a, clone 53-6.7 (11-

0081-85); eFluor-450 anti-mouse CD11b, clone M1/70 (48-0112-82); APC anti-mouse CD44, clone IM7 (47-0441-82); PerCP/Cy5.5 anti-mouse CD62L, clone MEL-14 (45-0621-82); PE anti-mouse F4/80, clone BM8 (12-4801-82); eFluor-450 anti-mouse NK1.1, clone PK136 (48-5941-82). Invitrogen (Waltham, MA): FITC anti-mouse cytokeratin, clone C-11 (MA5-28561).

Antibodies (Immunofluorescence/Immunoblotting) Primary antibodies included rabbit anti-HIF-1α (GeneTex, GTX30647), rabbit anti-β-actin (Clone AC-74, Sigma-Aldrich, cat. A2066), rat anti-mouse PD-L1 (Clone 10F.9G2, BioXCell, cat. BE0101) and rat anti-mouse CD3 (Clone CD3-12, Abcam, cat. ab11089). For immunofluorescence, Alexa Fluor® 594 goat anti-rat IgG (Invitrogen, cat. A11007) was used as the secondary antibody.

Reagents Phorbol 12-myristate 13-acetate (PMA), ionomycin, FITC-dextran, Collagenase Type IV, DNase Type IV, Hyaluronidase Type V, and Dispase Type II were from Sigma-Aldrich. Collagenase Type II was from Gibco. RPMI-1640, DPBS without calcium and magnesium (1xPBS) were from either Gibco or Corning. Red blood cell lysis buffer concentrate was from BD Bioscience (cat. BD 555899). Live/Dead™ Fixable Aqua Dead Cell Stain and eBioscience™ Foxp3/Transcription Factor Staining Buffer Set were from Life Technologies. BD Cytometric Bead Array (CBA) Mouse Inflammation Kit and GolgiStop™ were from BD Biosciences, and Recombinant Murine IFN-γ was from PeproTech.

Immunoblotting Cultured cells were lysed using 2% SDS lysing buffer and sonicated at 20A for 30 seconds prior to centrifugation and addition of 6x loading buffer. Samples were separated using 10% SDS-PAGE gel and transferred onto polyvinylidene fluoride (PVDF) membrane. The

membrane was blotted with primary antibody at 4°C overnight. After washing with 1xPBS containing 0.2% Tween 20, the membranes were blotted with secondary antibodies at room temperature for 3 hours, and developed with GE Amersham ECL prime solution.

Isolation and Processing of Tumor or Spleen Cells Following euthanasia, the tumors and/or spleens were dissected from the mice and placed on ice. Spleens were gently dissociated into petri dishes using frosted microscope slides and 10 ml RPMI-1640. To obtain single cell suspensions of tumors, the tumors were minced with scissors and digested at 37°C with 300 rpm shaking for 45 minutes in a triple-enzyme digestion buffer containing Collagenase Type IV (1 mg/ml), DNase Type IV (20 mg/ml), and Hyaluronidase Type V (0.1 mg/ml) in 1xPBS supplemented with 10% FBS. The digestion buffer concentrate was prepared at 10x and stored at -80°C and was thawed at room temperature immediately before use, wherein it was diluted in 1xPBS with 10% FBS. Subsequently, dissociated tumor or spleen cells were pelleted at 450 g for 5 minutes, resuspended in 1 ml 1x BD Lysing Buffer, and then washed with staining buffer (SB) consisting of 1xPBS supplemented with 10% FBS, 1 mM EDTA. The cells were then passed through 100 µm cell strainers, re-pelleted, and either directly stained for flow cytometry, or alternatively cultured for T cell stimulation and stained thereafter, as detailed in the following sections.

T Cell Stimulation and Intracellular Staining Spleen or tumor cells were resuspended with RPMI-1640 medium containing 10% FBS and 2% penicillin/streptomycin, and 1 µg/ml each of phorbol 12-myristate 13-acetate (PMA) (Sigma-Aldrich, St. Louis, MO), ionomycin (Sigma-Aldrich, St. Louis, MO) and BD GolgiStop™ (BD Biosciences, cat. 51-2092KZ), and

subsequently incubated for 4 hours at 37°C in 24-well or 48-well plates. Then, the cells were resuspended by pipette, transferred into Eppendorf tubes, washed with 1xPBS, and stained as detailed in the flow cytometry section.

Flow Cytometry For surface staining, cells were first washed in 1xPBS, resuspended in 1xPBS with 1x Live/Dead™ Fixable Aqua Dead Cell Stain (Life Technologies, Frederick, MD), and left on ice for 5 minutes to label dead cells. Then, the cells were washed in SB, and stained for 30 minutes on ice with antibodies at 1:100 dilution in SB. In certain cases, intracellular staining of IFNγ and/or Foxp3 (1:100 dilution) was further carried out on surface-stained cells with eBioscience™ Foxp3/Transcription Factor Staining Buffer Set (Life Technologies, Frederick, MD), according to the manufacturer's instructions. All data was collected on a BD FACS Canto II flow cytometer and subsequently analyzed using FlowJo v10.6.1 software.

Human CTLA4 knockin model On day 10 after birth, male and female $CTLA4^{h/h}$ mouse pups were fairly redistributed into different treatment groups based on sex and starting weight. To avoid cage variation, mice in the same cages and litters were individually tagged and allocated to different treatment groups. All test articles were administered by i.p. injection, starting on day 10 after birth. Echinomycin, or equivalent of empty liposomes, was administered at 10 µg/kg on days 10, 13, 16, 19, 22, 25, 28, and 31 after birth. Therapeutic antibodies, at 0.1 mg/mo. (Ipilimumab) or 0.2 mg/mo. (RMP1-14 or XMG1.2) were administered on days 10, 13, 16, and 19. After every treatment, the mice were returned to their mothers up until day 21, after which the mice were weaned and housed separately based on gender. On day 32, FITC-dextran assay was performed on all of the mice according to a previously described protocol (Gupta and

Nebreda, 2014). Briefly, 40 kDa FITC-dextran (Sigma-Aldrich, St. Louis, MO) was administered by oral gavage at 50 mg/kg, and blood sampling was performed 4 hours later by submandibular bleeding. The undiluted blood was collected using EDTA as anti-coagulant, and the plasma was retrieved thereafter by pelleting the samples at 450 g for 5 minutes. The plasma was subsequently diluted 1:3 in 1xPBS, and the fluorescence intensity was measured using excitation/emission of 485/528 nm with a SpectraMax iD3 plate reader (Molecular Devices, San Jose, CA). On day 33, the mice were euthanized, perfused with 1xPBS, and the tissues were handled for flow cytometry or histological analyses as described in the corresponding sections.

Determination of Intestinal IFNγ Levels To measure IFNγ in the intestinal tissues of *CTLA4*$^{h/h}$ mice, laparotomies were performed on the euthanized mice on day 33 after birth to expose the small intestine. The tissues were dissected by first transecting the pyloric sphincter to separate the stomach from small intestine, followed by a second incision at the duodenojejunal flexure. Each of the extracted duodenal tissues, roughly 0.2 g each, was individually weighed and minced with scissors. Then, 0.5 ml of intestinal digestion buffer (RPMI-1640 medium supplemented with 10% FBS plus 0.5 mg/ml dispase II and 1.5 mg/ml collagenase II) was added to each tissue in an Eppendorf tube, and digestion was carried out at 37°C with shaking at 250 rpm for 45 minutes. The digested tissues were then pelleted at 1000 g for 5 minutes and the supernatant was extracted, transferred into a new tube, and either stored at -80°C or used immediately. For analysis, the supernatant was sampled using a BD Cytometric Bead Array (CBA) Mouse Inflammation Kit (BD Biosciences, San Jose, CA) according to the manufacturer’s instructions, and the concentration of IFNγ was extrapolated using a standard curve and then normalized based on the weight of the intestinal tissue collected.

Histology and Immunofluorescence Mice were perfused with 1xPBS and the tissues were fixed in 10% neutral buffered formalin. After fixation, the tissues were trimmed, paraffin-embedded, sectioned, and hematoxylin and eosin (H&E) stained by Histoserv, Inc. (Gaithersburg, MD). Pathological examinations of H&E stained slides were performed blinded by a Histoserv, Inc. licensed veterinary pathologist, who scored and photographed H&E tissues accordingly. Additional unstained slides were returned for immunofluorescence analysis, which was performed in-house. For immunofluorescence, the unstained slides were sequentially deparaffinized and rehydrated, and antigen-retrieval was performed by microwave-boiling for 20 minutes in 0.5 M Tris-HCl plus 5% urea, pH 9.5. Slides were then methanol-fixed and permeabilized by 0.3% Triton, stained with antibodies in 2% bovine serum albumin blocking buffer, and lightly counterstained with 4',6-diamidino-2-phenylindole (DAPI) to visualize nuclei. After mounting, the slides were analyzed with an Olympus BX53 Light Microscope equipped with DP80 Dual CCD camera and CellSens Imaging software. For the scoring of T cell infiltration in kidney and liver, a graded scale was used as follows: 0, Normal/none; 1, minimal; 2, mild; 3, moderate; 4, severe.

HIF-1α and Pdl1shRNA knockdown ShRNA knockdown of Hif-1α, and PD-L1 was performed by introducing lentivirus, which is made with respective shRNA plasmid cocktails (one sh-RNA plus two package plasmids) in HEK293FT cells. Mixed lentiviruses of two distinct shRNA sequences were used to infect tumor cells for 72 hours before sorting fluorescence reporter or selecting drug-resistant transfected cells. The two lentiviral shRNA plasmids were constructed by cloning DNA oligo sequences of HIF-1α (sh1, 5-ggacaagtcaccacaagga; sh2, 5-

gaaactcaagcaactgtca), PD-L1-sh (sh1, 5- gaatcacgctgaaagtcaa; sh2, 5-aagatgagcaagtgattca), or scrambled sh (Sr-sh) (sh1, 5-gtgctatcacctcactgaa; sh2,5-gacatctcgacgtgcagcaa) in a lentiviral shRNA vector with GFP as a reporter (Wang et al., 2011). Puromycin selected shRNA fluorescence-positive tumor cells were cultured to reach required numbers for in vitro treatment or injected subcutaneously into fat pad of recipient mice, ($1x10^6$/mouse).

HRE Reporter Assay A reporter system for HIF activity in tumor cells was established by our group and reported previously (Wang et al., 2011). Briefly, Hif-1α-specific reporter plasmid was made by cloning three tandem repeats of Hypoxia response element (HRE) (5'-TCTGTACGTGACCACACTCACCTC-3') into a lentiviral vector, in which HREs locate upstream TATA-driven GFP reporter and downstream promoter EF1a-driven puromycin. Cells were infected with a reporter system lentivirus, and 48 hours later, cells were passaged. After overnight, puromycin (2ug/ml) was added and puromycin resistant cells were used for the treatments. Statistics analyzed by one-way ANOVA with Sidak's posttest.

Full unedited gel for Figure 1A

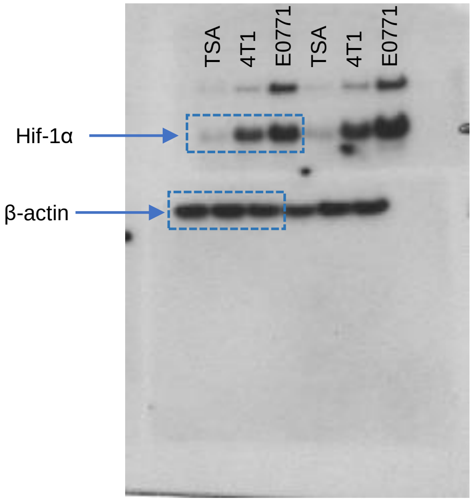

Supplement: Supplemental data [file jci-132-150846-s101.pdf]
